# Significant loss of soil inorganic carbon at the continental scale

**DOI:** 10.1093/nsr/nwab120

**Published:** 2021-07-02

**Authors:** Xiao-Dong Song, Fei Yang, Hua-Yong Wu, Jing Zhang, De-Cheng Li, Feng Liu, Yu-Guo Zhao, Jin-Ling Yang, Bing Ju, Chong-Fa Cai, Biao Huang, Huai-Yu Long, Ying Lu, Yue-Yu Sui, Qiu-Bing Wang, Ke-Ning Wu, Feng-Rong Zhang, Ming-Kui Zhang, Zhou Shi, Wan-Zhu Ma, Gang Xin, Zhi-Ping Qi, Qing-Rui Chang, En Ci, Da-Gang Yuan, Yang-Zhu Zhang, Jun-Ping Bai, Jia-Ying Chen, Jie Chen, Yin-Jun Chen, Yun-Zhong Dong, Chun-Lan Han, Ling Li, Li-Ming Liu, Jian-Jun Pan, Fu-Peng Song, Fu-Jun Sun, Deng-Feng Wang, Tian-Wei Wang, Xiang-Hua Wei, Hong-Qi Wu, Xia Zhao, Qing Zhou, Gan-Lin Zhang

**Affiliations:** State Key Laboratory of Soil and Sustainable Agriculture, Institute of Soil Science, Chinese Academy of Sciences, Nanjing 210008, China; State Key Laboratory of Soil and Sustainable Agriculture, Institute of Soil Science, Chinese Academy of Sciences, Nanjing 210008, China; State Key Laboratory of Soil and Sustainable Agriculture, Institute of Soil Science, Chinese Academy of Sciences, Nanjing 210008, China; State Key Laboratory of Soil and Sustainable Agriculture, Institute of Soil Science, Chinese Academy of Sciences, Nanjing 210008, China; College of Advanced Agricultural Sciences, University of Chinese Academy of Sciences, Beijing 100049, China; State Key Laboratory of Soil and Sustainable Agriculture, Institute of Soil Science, Chinese Academy of Sciences, Nanjing 210008, China; State Key Laboratory of Soil and Sustainable Agriculture, Institute of Soil Science, Chinese Academy of Sciences, Nanjing 210008, China; State Key Laboratory of Soil and Sustainable Agriculture, Institute of Soil Science, Chinese Academy of Sciences, Nanjing 210008, China; College of Advanced Agricultural Sciences, University of Chinese Academy of Sciences, Beijing 100049, China; State Key Laboratory of Soil and Sustainable Agriculture, Institute of Soil Science, Chinese Academy of Sciences, Nanjing 210008, China; College of Advanced Agricultural Sciences, University of Chinese Academy of Sciences, Beijing 100049, China; State Key Laboratory of Soil and Sustainable Agriculture, Institute of Soil Science, Chinese Academy of Sciences, Nanjing 210008, China; College of Resources and Environment, Huazhong Agricultural University, Wuhan 430070, China; State Key Laboratory of Soil and Sustainable Agriculture, Institute of Soil Science, Chinese Academy of Sciences, Nanjing 210008, China; Institute of Agricultural Resources and Regional Planning, Chinese Academy of Agricultural Sciences, Beijing 100081, China; College of Natural Resources and Environment, South China Agricultural University, Guangzhou 510642, China; Northeast Institute of Geography and Agroecology, Chinese Academy of Sciences, Harbin 150081, China; College of Land and Environment, Shenyang Agricultural University, Shenyang 110161, China; School of Land Science and Technology, China University of Geosciences, Beijing 100083, China; College of Land Science and Technology, China Agricultural University, Beijing 100193, China; College of Environmental and Resource Sciences, Zhejiang University, Hangzhou 310058, China; College of Environmental and Resource Sciences, Zhejiang University, Hangzhou 310058, China; Institute of Digital Agriculture, Zhejiang Academy of Agricultural Sciences, Hangzhou 310021, China; College of Agriculture, Heilongjiang Bayi Agricultural University, Daqing 163319, China; Tropical Crops Genetic Resources Institute, Chinese Academy of Tropical Agricultural Sciences, Haikou 571101, China; College of Natural Resources and Environment, Northwest A & F University, Yangling 712100, China; College of Resources and Environment, Southwest University, Chongqing 400715, China; College of Resources, Sichuan Agricultural University, Chengdu 611130, China; College of Resources and Environment, Hunan Agricultural University, Changsha 410128, China; Institute of Agricultural Product Quality Standard and Testing Research, Tibet Academy of Agricultural and Animal Husbandry Sciences, Lhasa 850032, China; College of Resources and Environment, Huazhong Agricultural University, Wuhan 430070, China; School of Agricultural Sciences, Zhengzhou University, Zhengzhou 450001, China; Institute of Agricultural Resources and Regional Planning, Chinese Academy of Agricultural Sciences, Beijing 100081, China; Institute of Agriculture Environment and Resources Research, Shanxi Academy of Agricultural Sciences, Taiyuan 030006, China; College of Land and Environment, Shenyang Agricultural University, Shenyang 110161, China; College of Resources and Environment, Henan Agricultural University, Zhengzhou 450002, China; College of Resources and Environment, China Agricultural University, Beijing 100193, China; College of Resources and Environmental Sciences, Nanjing Agricultural University, Nanjing 210095, China; College of Resources and Environment, Shandong Agricultural University, Taian 271018, China; College of Land and Environment, Shenyang Agricultural University, Shenyang 110161, China; Tropical Crops Genetic Resources Institute, Chinese Academy of Tropical Agricultural Sciences, Haikou 571101, China; College of Resources and Environment, Huazhong Agricultural University, Wuhan 430070, China; Agricultural College, Guangxi University, Nanning 530005, China; College of Grassland and Environment Science, Xinjiang Agricultural University, Urumqi 830052, China; College of Geographical Science, Qinghai Normal University, Xining 810008, China; College of Resources and Environment, Hunan Agricultural University, Changsha 410128, China; State Key Laboratory of Soil and Sustainable Agriculture, Institute of Soil Science, Chinese Academy of Sciences, Nanjing 210008, China; College of Advanced Agricultural Sciences, University of Chinese Academy of Sciences, Beijing 100049, China; Key Laboratory of Watershed Geographic Sciences, Nanjing Institute of Geography and Limnology, Chinese Academy of Sciences, Nanjing 210008, China

**Keywords:** China, soil inorganic carbon stocks, global change, carbonate, soil acidification

## Abstract

Widespread soil acidification due to atmospheric acid deposition and agricultural fertilization may greatly accelerate soil carbonate dissolution and CO_2_ release. However, to date, few studies have addressed these processes. Here, we use meta-analysis and nationwide-survey datasets to investigate changes in soil inorganic carbon (SIC) stocks in China. We observe an overall decrease in SIC stocks in topsoil (0–30 cm) (11.33 g C m^–2^ yr^–1^) from the 1980s to the 2010s. Total SIC stocks have decreased by ∼8.99 ± 2.24% (1.37 ± 0.37 Pg C). The average SIC losses across China (0.046 Pg C yr^–1^) and in cropland (0.016 Pg C yr^–1^) account for ∼17.6%–24.0% of the terrestrial C sink and 57.1% of the soil organic carbon sink in cropland, respectively. Nitrogen deposition and climate change have profound influences on SIC cycling. We estimate that ∼19.12%–19.47% of SIC stocks will be further lost by 2100. The consumption of SIC may offset a large portion of global efforts aimed at ecosystem carbon sequestration, which emphasizes the importance of achieving a better understanding of the indirect coupling mechanisms of nitrogen and carbon cycling and of effective countermeasures to minimize SIC loss.

## Teaser

Significant loss of soil inorganic carbon forced by the input and transformation of anthropogenic reactive nitrogen at the continental scale offsets much of carbon sequestration in the terrestrial ecosystems.

## INTRODUCTION

In the top 2 m of soil, global soil organic carbon (SOC) and soil inorganic carbon (SIC) stocks have been estimated to be 1993 Pg C [1] and >2300 Pg C [2], respectively. Recent studies have shown that the Earth is becoming more vegetated and that biological carbon sequestration is significant [3]. Similarly, individual countries (e.g. China) have seen SOC increases over the last three decades [4,5]. In contrast to SOC, SIC is forming slowly and is considered temporally stable [6]. Therefore, little attention has been paid to SIC pool dynamics. Nonetheless, the most recent studies show that environmental changes, such as the use of chemical fertilizers, global warming and atmospheric acid deposition, have caused significant global soil acidification, especially in cropland [7,8], which may accelerate SIC turnover [9,10], leading to SIC sinks [11,12] or SIC sources [13,14]. Land use change may also influence SIC cycling [15].

Most SIC (up to 90%) contributes to the total soil C stock in arid and semi-arid areas [15,16], which account for ∼41% of the Earth's land surface [17,18]. Over two-fifths of China's land is covered by arid and semi-arid biomes (∼4.3 million km^2^) [19]. As the second largest economy and the most populous country, China has experienced rapid land use changes [20], prominent afforestation [3] and immensely intensified agriculture by ever-increasing investment in chemical fertilizers [4]. Many environmental policies and laws have been enacted to protect terrestrial ecosystems [21]. The effect of these drivers on soil C dynamics has been widely reported for SOC [4,5] but rarely reported for SIC, despite pronounced soil acidification across China's cropland [7], forestland [22] and grassland [23]. Due to widespread soil acidification, changes in SIC may release massive amounts of C and offset SOC sequestration in China [4,5,20]. However, reported results at the regional or national scale were obtained using observational data from the Second National Soil Survey in the 1980s [14,24,25] and lacked present-day soil data. Despite its recognized importance, the spatiotemporal variation in the SIC pool across China in recent decades remains uncertain and incomplete due to data limitation.

## RESULTS AND DISCUSSION

### Historic changes in SIC

To fill the knowledge gap, we conducted a national soil resampling campaign (2009–2019) and compared data with legacy data from previous soil surveys to evaluate the changes in SIC stocks (0–30 cm) across China's cropland, forest and grassland (Supplementary Text and Supplementary Fig. S1). The study area spans from near-central Asia to the eastern part of the Eurasian continent. The soil dataset includes three subsets from 13 769 sites spanning the 1980s, 2000s and 2010s. The national soil data in the 1980s were taken as control plots. Soil data in the 2000s were synthesized from publications for validation.

**Figure 1.**
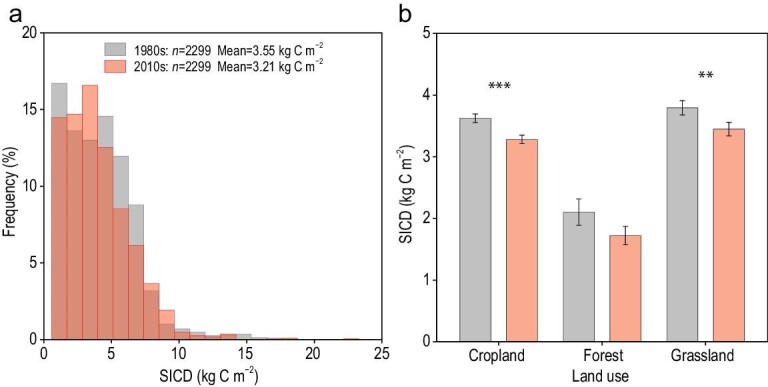
Changes in soil inorganic carbon density (SICD) at soil depths of 0–30 cm from the 1980s to the 2010s across China. (a) Histograms of observations. (b) Mean values of observations across cropland (*n *= 1528), forest (*n *= 177) and grassland (*n *= 594) areas. A paired *t*-test was used to evaluate the differences. Soil points in the 1980s were taken as control samples, and soil samples without carbonate were excluded. ^**^ indicates *P *< 0.01, and ^***^ indicates *P *< 0.001. The error bars represent the standard errors. Their distributions are normal after square-root transformation.

**Figure 2.**
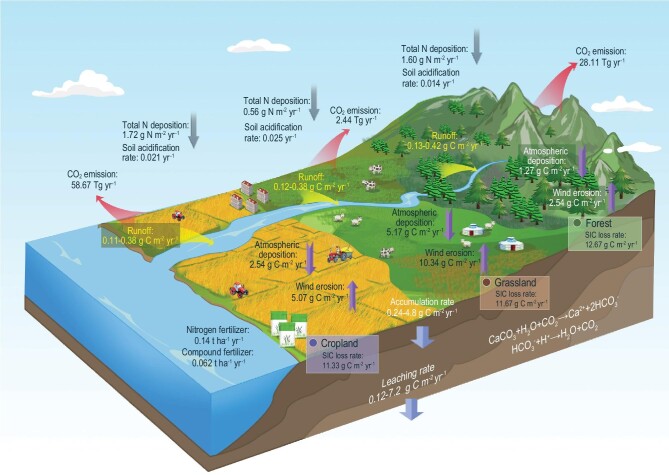
SIC dynamics in China and their main controls. The loss of topsoil SIC includes mainly runoff into the aquatic system, leaching to the subsoil, wind erosion and emissions of CO_2_ into the atmosphere. In contrast to natural ecosystems, agronomic advances can greatly affect the input/output of SIC by applying chemical fertilizers, especially nitrogen fertilizers. The accumulation and leaching rates of SIC are ranges for three ecosystems. These processes might be imbalanced, and the maximum total CO_2_ emissions are presented according to the spatial prediction (Supplementary Text) because not all of the inputs and outputs of SIC are illustrated; only the main processes are shown for clarity.

The overall change in SIC stocks (0–30 cm) across 2299 paired samples (Supplementary Text and Fig. 1) was significant based on the paired *t*-test (*P* < 0.001), and the mean values of SIC density (SICD) were 3.55 kg C m^–2^ and 3.21 kg C m^–2^ for the 1980s and 2010s, respectively. Compared with the control plots in the 1980s, ∼56.5% of the pairs exhibited a declining trend (1299/2299). The average SICD decrements across China's cropland, forest and grassland were 0.34 kg C m^–2^, 0.38 kg C m^–2^ and 0.35 kg C m^–2^, respectively. The mean declining rates for these three ecosystems were 11.33 g C m^–2^ yr^–1^, 12.67 g C m^–2^ yr^–1^ and 11.67 g C m^–2^ yr^–1^, respectively (Fig. 2). We also calculated the overall change in SIC stocks by using paired samples within a distance ranging from 10 km to 50 km (Supplementary Fig. S2). A net SIC loss was found in all cases. The declining trend was generally consistent with the unpaired *t*-test based on all the observations in the 1980s and 2010s, and the published data in the 2000s from the meta-analysis (Supplementary Fig. S3). Notably, the changes in SIC in the forestland were significant based on the unpaired comparison. The changes in SICD differed among the six agroecological zones (Supplementary Fig. S4). A significant decrease in SICD was found across China's cropland except in the northern and northeastern zones. SICD decreased significantly in the northern zone's forestland and the northern and northwestern zones’ grassland.

A net SIC loss was simultaneously shown by the paired (Fig. 1) and unpaired comparisons (Supplementary Fig. S3). In addition, we compared our results with those of recent studies on spatiotemporal changes in SIC (Supplementary Table S1). There was a disagreement between these case studies conducted in local geographical areas, suggesting that the accumulation and formation of SIC are complex, and the transformations among SOC, SIC and CO_2_ may greatly differ, particularly after land use change [15]. Even so, the present conclusion agrees with the studies conducted at the regional scales [14,25–27] and can provide an important observational benchmark for projecting the consequences of climate and land use change.

**Figure 3.**
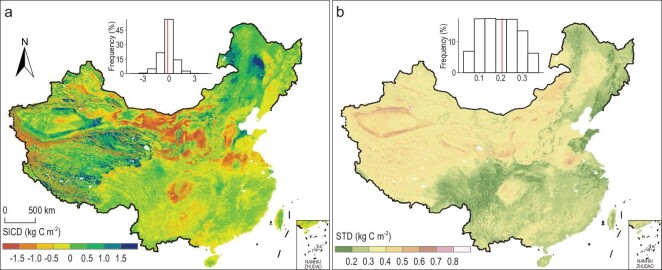
Spatial distribution of changes in topsoil (0–30 cm) SICD across China from the 1980s to the 2010s. (a) Mean changes in SICD for soil depths of 0–30 cm in the last three decades. (b) Standard deviations (STD) of changes in SICD based on 100 simulations. The inset is the relative frequency histogram of each map, in which the red line indicates the mean value.

The observations collected in the 1980s and 2010s were extrapolated to estimate the spatiotemporal changes in topsoil SIC stocks (Supplementary Text, Fig. 3 and Supplementary Fig. S5). Regarding the large computation requirement, we developed a new R package called ‘ParallelDSM’ [28] to perform the soil mapping in parallel. The random forest (RF) technique, with *R*^2^ values of 0.31 and 0.42 for the 1980s and 2010s, respectively, outperformed the other considered algorithms (Supplementary Fig. S6). Thus, only the RF method was employed rather than an ensemble method [29,30]. To assess the prediction uncertainty, the spatial distribution of the standard deviation (STD) of 100 simulations was generated (Fig. 3b and Supplementary Fig. S5b and d). High uncertainty in terms of high STD was found in some areas with high SICD (Fig. 3 and Supplementary Fig. S5). The spatial pattern of SICD showed a clear pattern in line with climatic and geological zones in the 1980s and 2010s, and the mean SICD values were 1.64 ± 0.14 kg C m^–2^ and 1.50 ± 0.14 kg C m^–2^, respectively. High SIC values were mainly found in the arid and semi-arid areas (North and Northwest China) (Supplementary Fig. S5). In the 1980s–2010s, total SIC stocks within the topsoil (30 cm) decreased by 8.99 ± 2.24% (1.37 ± 0.37 Pg C), approximately one-third of which was lost in the northwestern zone (Fig. 3 and Supplementary Table S2). All agroecological zones except the northeastern zone (where SIC stocks increased by 12.50 ± 4.71% (0.09 ± 0.03 Pg C)) have experienced net SIC losses. Specifically, the fastest loss of SIC was found in the south-central zone, with a value of 26.47 ± 4.03% (0.27 ± 0.05 Pg C), possibly due to the high lithogenic carbonate concentrations in the karst areas.

Three-dimensional (3D) soil mapping techniques were used to estimate total SIC stocks at a soil depth of 0–3 m in the 2010s (Supplementary Text). The depth functions accounted for 36%–89% of the vertical variations in SIC mass density (Supplementary Fig. S7), and thus, the missing SICD values were calculated down to a soil depth of 3 m. The RF model yielded the most accurate predictions for the estimates at 0–1 m, 1–2 m and 2–3 m (Supplementary Fig. S6). The produced SICD maps suggested that a mass of SIC was buried in the subsoil regardless of the ecosystem or agroecological zone (Supplementary Table S3). Total SIC stocks across China were 53.58 ± 0.57 Pg C, 134.96 ± 0.91 Pg C and 232.21 ± 1.21 Pg C for the upper 1 m, 2 m and 3 m, respectively. Of the SIC stocks, ∼47% were estimated in Northwest China, with 24.82 ± 0.37 Pg C, 62.52 ± 0.54 Pg C and 108.57 ± 0.62 Pg C for the upper 1 m, 2 m and 3 m, respectively. The SIC pool in the top 3 m for each zone varied among ecosystems (Supplementary Fig. S8). For example, cropland contributed primarily to the SIC pool in the eastern and northeastern zones (4.71 ± 0.05 Pg C and 5.68 ± 0.10 Pg C, respectively). High SIC values were predicted in forestland (4.28 ± 0.11 Pg C) in the south-central zone. In the other three zones, large amounts of SIC were stored in the grassland.

Reported SIC stocks at a soil depth of 1 m were consistent with the prediction using legacy data in the 1980s [24,25], and the SIC decreasing rates agreed with studies for various regions or ecosystems [25–27]. The changes in SIC stocks at 0–30 cm were greater than recent estimates [13,14]. This result could be attributed to the lack of direct observations in the 2010s, which may be the main source of uncertainty in those studies [13,14].

Across China's cropland, the increase rate of SOC stocks was 14 g C m^–2^ yr^–1^ in the 0–20 cm soil [4] and ∼21 g C m^–2^ yr^–1^ in the 0–30 cm soil by multiplying an expansion factor defined as depth ratios (30 cm/20 cm), approximately twice the loss rate of SIC (11.33 g C m^–2^ yr^–1^; Fig. 1). Notably, the overall changes in the SIC pool (0–30 cm) (Supplementary Table S2) were of the same magnitude as those in the national SOC sink at a depth of 1 m [5]. The average loss rate of SIC in forestland (7.7 ± 2.4 Tg C yr^–1^) was greater than that of sequestrated SOC (4.0 ± 4.1 Tg C yr^–1^), but the loss rates of SIC in cropland (16.0 ± 1.4 Tg C yr^–1^) and grassland (0.7 ± 1.3 Tg C yr^–1^) were lower than those of sequestrated SOC (26.0 ± 11.0 Tg C yr^–1^ for cropland and 6.0 ± 1.0 Tg C yr^–1^ for grassland). The average SIC losses across China (46 Tg C yr^–1^) and in cropland (16 Tg C yr^–1^) (Supplementary Table S2) account for ∼17.6%–24.0% of the terrestrial C sink [4] and 57.1% of the SOC sink in cropland [5], respectively. In addition, the potential of SOC accumulation remains unclear, but the results in the near future are not very optimistic [31,32]. In conclusion, the negative contribution of SIC to the soil C pool is far from negligible. To maintain the global terrestrial carbon balance, the feedback of SIC to global changes should be considered in the ongoing sixth assessment report of the Intergovernmental Panel on Climate Change and the ‘4 per 1000’ Initiative Strategic Plan [33].

### Linking SIC dynamics to intensified reactive N input and global change

The main pathways of SIC cycling based on our results and meta-analysis were determined in this study (Supplementary Text and Fig. 2). The pathway analysis showed that soil acidification may greatly accelerate SIC turnover, and thus global change, in terms of climate change, atmospheric nitrogen (N) deposition and anthropogenic reactive N addition indirectly lead to the loss of SIC (Fig. 2). Most of the lost SIC was converted to CO_2_ and released to the atmosphere (maximum of 89.22 Tg yr^–1^). The pathway analysis provided new insights into SIC pool dynamics and was helpful for understanding the spatial controls. Some non-essential C flows were not listed, such as sulfur deposition, manure application and plant uptake of Ca^2+^. The amount of manure application was much less than that of chemical fertilizer during the 1980s and 2000s in China [4]. The atmospheric inputs of Ca^2+^ mainly control pedogenic carbonate formation in arid areas (∼1.0–3.5 g CaCO_3_ m^–2^ yr^–1^) [34]. We did not calculate other processes affecting soil acidification [14] but used clear evidence from numerous observations in agroecosystems (*n *= 8875 in Ref. [7]) and natural ecosystems (*n *= 5598 in Ref. [22]; *n *= 602 in Ref. [23]).

We also conducted a data-driven analysis to identify the spatial controls on SIC loss. First, to rank the environmental controls, we measured relative variable importance through a Monte Carlo simulation of RF models (Supplementary Text and Supplementary Fig. S9). The results for the 1980s and 2010s showed that climatic variables and N deposition mainly drove the spatial pattern of SICD, such as the mean annual precipitation (MAP), mean annual temperature (MAT) and total NH*_x_* and NO*_y_* deposition. Furthermore, the correlations between the changes in SICD and those in climate and N deposition were analyzed. Despite the absence of a strong correlation, the changes in SICD in different ecosystems varied markedly with the changes in these variables (*P *< 0.05) (Supplementary Fig. S10). Much SIC will be lost due to increasing MAP, NH_*x*_ and NO_*y*_.

However, the actual amount of net CO_2_ emission resulting from carbonate dissolution was difficult to account for, as Ca^2+^ ions were not often recorded in either period. The exchangeable calcium (Exch. Ca) may increase due to the dissolution of carbonates [35], some of which may leach downward along with NO_3_^–^ and HCO_3_^–^ [36]. Here, Exch. Ca data from four provinces, i.e. Hubei, Henan, Sichuan and Guangdong provinces, in the south-central and southwestern zones, were collected. The results of the unpaired *t*-test showed that Exch. Ca significantly increased by 2.12 cmol (+) kg^–1^ and 1.72 cmol (+) kg^–1^ in cropland and forestland, respectively (Supplementary Table S4). These results suggested that more CaCO_3_ was dissolved than before, even if the increase in Exch. Ca in cropland can be partly attributed to fertilization (e.g. calcium-magnesium phosphate).

In conclusion, global changes as aggravated by the anthropogenic input of reactive N via direct or indirect pathways were the main drivers of SIC loss according to three different methods: pathway analysis (Fig. 2), variable importance analysis (Supplementary Fig. S9) and correlation analysis (Supplementary Fig. S10). In theory, to achieve a net loss of SIC, the SIC input should be less than the output. Changes in Ca^2+^, H^+^ and HCO_3_^–^ concentrations, soil water content and CO_2_ pressure can directly stimulate the equilibrium reaction of carbonate dissolution (equation in Fig. 2) [37]. Hence, the driving factors inferred above could be briefly explained as follows. First, in the last three decades, intensified agriculture consumed large amounts of chemical N fertilizer (∼0.135 ton ha^–1^ yr^–1^) (Supplementary Fig. S11), and anthropogenic activities caused a rapid increase in atmospheric N deposition [38]. Thus, nationwide soil acidification was observed. A decrease in SIC stocks resulting from net H^+^ input likely occurred [14,27]. Carbonate dissolution was greatly accelerated by neutralizing soil acidification, especially in calcareous soils with pH > 6.5 [14,22]. Second, the increase in atmospheric CO_2_ concentrations may directly elevate CO_2_ in topsoil, and global warming is even expected to increase CO_2_ production from entire soil profiles [39]. The average precipitation has increased over mid-latitude land areas since 1901 [40], leading to an increase in soil water content. Finally, the agroecosystem exhibited a net SOC sink due to the fast increase in crop production and return of crop residues to cropland [4]. The Chinese government has issued a series of laws, regulations and policies to alleviate environmental problems, such as the Natural Forest Conservation Program and Grain for Green Program [21]. These strategies are very effective. For example, China contributed to 25% of the global increase in leaf area [3], and SOC was obviously sequestered (75.0–75.4 Tg C yr^–1^) in China [5]. The terrestrial biosphere C sink may offset 45% of annual Chinese anthropogenic C emissions [41]. Accordingly, the accumulation of SOC may promote organic and carbonic acid production, soil structure, soil porosity, and heterotrophic and autotrophic respiration [37], which often neutralize base cations, enhance CO_2_ partial pressures and soil water content, and facilitate the leaching and dissolution of carbonate [15]. Therefore, this study provides evidence of a trade-off between SOC and SIC stocks, which is similar to the trade-off between plant biomass and SOC stocks resulting from elevated CO_2_ [42].

### Future projection

**Figure 4.**
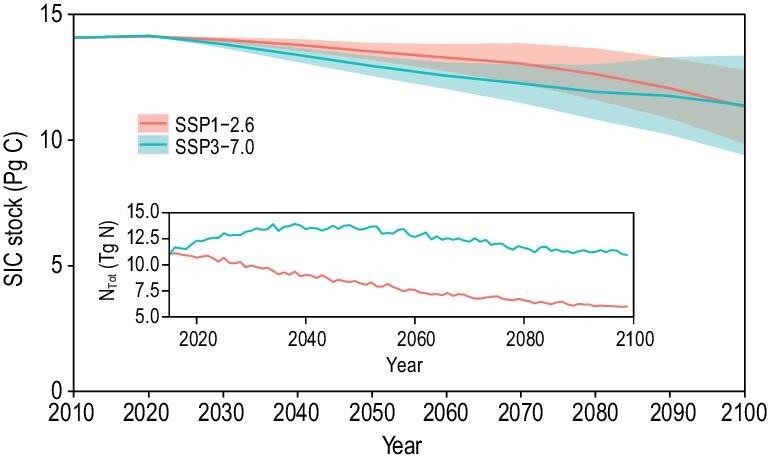
Trend in topsoil (0–30 cm) SIC stocks in China for the 2010–2100 period under the SSP1–2.6 and SSP3–7.0 scenarios. The solid lines represent the mean values, and the shaded regions indicate all the predicted values from nine CMIP6 models. The prediction uncertainty for each scenario was not assessed because of the large calculation cost. The changes in total N deposition (N_Tot_) are illustrated in the inset, and were obtained from Ref. [44]. The time steps for the SIC stocks modeling are the decades from 2010 to 2100, aiming to better quantify the cumulative effect of environmental changes on SIC.

The formation rates of both lithogenic and pedogenic inorganic carbon are generally lower than the neutralizing rates for increasing acidity [6,13,36]. Under future climate scenarios, SIC stocks are expected to continue decreasing if more protons are released into soils. We evaluated the potential risk of SIC loss from 2020 to 2100 (Supplementary Text), in which the 2010s subset was taken as the baseline, mainly driven by land use and climate projections from nine Coupled Model Intercomparison Project Phase 6 (CMIP6) models under Shared Socioeconomic Pathways (SSPs) 1–2.6 and 3–7.0 [43]. Total topsoil SIC stocks by the end of this century were projected to be 11.33 ± 1.47 Pg C and 11.38 ± 1.98 Pg C for SSP1–2.6 and SSP3–7.0, respectively (Fig. 4). Compared to the 2010s, SIC was estimated to decrease by 6.45%–40.05% and 7.21%–52.37% for SSP1–2.6 and SSP3–7.0, with mean values of 19.47% and 19.12%, respectively. As mentioned above, most of the SIC may be released to the atmosphere as CO_2_ (Fig. 2). This trend will continue under two SSP scenarios through 2100 (Fig. 4). Approximately one-fifth of the SIC will be released to the atmosphere as CO_2_. The uncertainty in the aforementioned ranges is approximately double (SSP1–2.6) or triple (SSP3–7.0) the mean values. This result showed that different future climate scenarios may lead to moderate projection uncertainty.

The SIC in cropland will decrease by 31.27% (SSP1–2.6) or 52.90% (SSP3–7.0), while the SIC in natural ecosystems (forest and grassland) may decrease by 12.82% (SSP1–2.6) or 50.52% (SSP3–7.0). The projection shows that under SSP1–2.6, SIC is anticipated to stop decreasing in natural ecosystems (Supplementary Fig. S12b). This is a positive sign and indicates that stakeholders and decision makers can take environmentally friendly actions to avoid dangerous anthropogenic interference with the climate system. In this century, it is very likely that global climate change will be gradually reinforced [40]. Linking these results together, it can be inferred that global change will continue to facilitate SIC loss. The common continuous loss of SIC is far from negligible and should be considered in the global biogeochemical cycling of carbon.

Although the RF model provided a robust prediction (Supplementary Fig. S6), uncertainty was inevitably present in the SIC pool estimation. Due to the lack of accurate longitudinal and latitudinal information on legacy points, the repeated sampling locations in the 2010s were not completely identical to those in the 1980s. In addition, differences in the number and location of collected soil samples (Supplementary Fig. S1) may have led to variation in the results, as soils with carbonates were mainly found in arid and semi-arid regions, or in the karst areas. For example, the observed SICD values in cropland (Fig. 1) were moderately greater than those in Refs [14,45], and the SICD values in grassland were slightly less than those in Ref. [46]. The average loss rates of SIC in cropland were greater than those in Ref. [14], and those in grassland were less than those in Ref. [27]. However, estimated SIC stocks at soil depths of 0.3 m and 1 m (Supplementary Tables S2 and S3) were consistent with the predictions of Refs [45] and [24,25,45], respectively. The conclusions of net SIC loss in different ecosystems (Fig. 1 and Supplementary Fig. S4) concurred with the findings of studies conducted at the regional scale [2,14,25–27], which can provide credible observational benchmarks.

Furthermore, the mechanism of carbonate dissolution resulting from N input in various ecosystems and the pathways of Ca^2+^ ions remain unclear (Fig. 2). The dissolution, precipitation and leaching of carbonate may be jointly responsible for SIC losses [35]. Therefore, CO_2_ emissions that are due to acidifying processes need to be accurately quantified [13,14]. In addition, the future projections of SIC change in this study relied mainly on the parameters of the baseline model in the 2010s and dynamic variables (Supplementary Text). Thus, the prediction errors of the baseline model might have been propagated when incorporating future environmental variables as covariates, which were characterized by inherent errors from production and downscaling. For example, although annual N deposition may moderately decrease in SSP1–2.6, the cumulative decrease in total SIC stocks is similar to that in SSP3–7.0 (Fig. 4). Similar changes are also found in agroecosystems (Supplementary Fig. S12). The uncertainty of the future projection was not assessed because of the large calculation cost. However, it can be inferred that the prediction uncertainty from 2020–2100 would be of the same magnitude as that in the 2010s (Supplementary Table S2), which is acceptable.

## CONCLUSION

A net SIC loss of 0.046 Pg C yr^–1^ is found in China from the 1980s to the 2010s. Spatial analysis shows that N deposition and climate change have profound influences on SIC loss. Assuming these influences continue, under the low and medium/high forcing pathways of the CMIP6 models, ∼19.12%–19.47% of the SIC stocks in China will be further lost by the end of this century. We conclude that the consumption of SIC may offset a large portion of global efforts aimed at SOC sequestration and the overall ecosystem carbon sink, which highlights how important it is to better understand the complex coupling mechanisms of nitrogen and carbon cycling and to provide solutions to mitigate further SIC loss.

## METHODS

Detailed methods are given in the online supplementary material.

## Supplementary Material

nwab120_Supplemental_File
