# Supplementary material for: Significant loss of soil inorganic carbon at the continental scale
Source: Natl Sci Rev. 2021 Jul 2;9(2):nwab120. doi: 10.1093/nsr/nwab120 (PMC8824702; doi:10.1093/nsr/nwab120)
Supplement: nwab120_Supplemental_File [file nwab120_supplemental_file.pdf]

Supplementary Materials for

Significant loss of soil inorganic carbon at the continental scale

Xiao-Dong Song, Fei Yang, Hua-Yong Wu, Jing Zhang, De-Cheng Li, Feng Liu, Yu-Guo Zhao, Jin-Ling Yang, Bing Ju, Chong-Fa Cai, Biao Huang, Huai-Yu Long, Ying Lu, Yue-Yu Sui, Qiu-Bing Wang, Ke-Ning Wu, Feng-Rong Zhang, Ming-Kui Zhang, Zhou Shi, Wan-Zhu Ma, Gang Xin, Zhi-Ping Qi, Qing-Rui Chang, En Ci, Da-Gang Yuan, Yang-Zhu Zhang, Jun-Ping Bai, Jia-Ying Chen, Jie Chen, Yin-Jun Chen, Yun-Zhong Dong, Chun-Lan Han, Ling Li, Li-Ming Liu, Jian-Jun Pan, Fu-Peng Song, Fu-Jun Sun, Deng-Feng Wang, Tian-Wei Wang, Xiang-Hua Wei, Hong-Qi Wu, Xia Zhao, Qing Zhou and Gan-Lin Zhang*

Correspondence to: glzhang@issas.ac.cn

This file includes:

Supplementary Text: Materials and Methods

Supplementary Figures: Figs. S1 to S12

Supplementary Tables: Tables S1 to S7

Supplementary Text

Materials and Methods

Soil data

Soil samples were gathered from 13769 sites across mainland China in the 1980s (n=7299), 2000s (n=774) and 2010s (n=5696) (Supplementary Fig. S1). Soil data in the 1980s were manually digitalized from 30 hard-copy monographs (Supplementary Table S5) of the Second National Soil Survey (1979-1984). This subset might be the most comprehensive legacy soil data [1-3], in which detailed pedological information and physicochemical properties were recorded. Soil data for the islands of the South China Sea, Macao, Hong Kong and Taiwan were not available.

We conducted the national field resampling campaign from 2009 to 2019 across different ecosystems and collected 23535 soil samples [4]. For the soil sampling design, the soil pedons in the 1980s were taken as control plots. The sampling locations were mainly guided by the sites and soil-forming information of the 1980s' points to detect the spatiotemporal soil variation. Therefore, the sampling sites in the 2010s were located adjacent to the sampling sites in the 1980s. A 1×2 m plot was laid out before digging, and soil profiles were dug to 1 m soil depth or to fresh bedrock (< 1 m). Soil samples (~1 kg) of each horizon were taken. Morphologic features of genetic horizons, as well as photos of soil profiles and corresponding landscapes were recorded in the field according to the Soil Taxonomy guidelines. The soil samples collected across different ecosystems differed in their edaphic properties.

We compiled soil inorganic carbon (SIC) data from publications from 2000 to 2010 via "China National Knowledge Infrastructure" (https://www.cnki.net/) and "Web of Science" (http://www.isiknowledge.com/). Various keywords related to this study were used, such as "China", "cropland", "grassland", "forest", "soil inorganic carbon", "carbonate", "deep soil", "arid soil" and "soil taxonomy". A total of 145 articles and dissertations were screened from 75 publications, and 774 average values were obtained from 3,612 sampling sites (Supplementary Table S6). If the soil sampling time was not given, the manuscript submission time was used. The median time was employed if the sampling campaign was conducted over multiple years. Soil data in graphs were digitalized with ScanIt 2.0.7 (AmsterCHEM, Netherlands). Since carbonate was not always found in noncalcareous soils, SIC data were rarely reported in publications. Therefore, a linear function (R^2=0.88) was used to convert SIC data measured by different methods [5].

After air-drying, soil samples were sieved through a 2-mm mesh. The carbonates include $MgCO_3$, $CaCO_3$ and other carbonate minerals, and $CaCO_3$ is usually dominant [6]. $CaCO_3$ concentrations of the 1980s and 2010s subsets were measured with acid dissolution (pressure calcimeter method) [2]. A coefficient of 0.12 was used to convert the $CaCO_3$ concentration to SIC. Bulk density (BD) was analyzed with the core method. SIC density (SICD; kg C m^{-2}) was computed by aggregating SIC concentration (g kg^{-1}), BD (g cm^{-3}), the volume of coarse fragment (Gr) (%), and thickness (Th, m) in each soil layer:

$$SICD = SIC \times BD \times (1\text{-}Gr) \times Th. \quad (1)$$

The sum of SICD in the 0-30 cm soil was used to represent topsoil SICD at a site.

The BD data were unavailable for many samples, and several published linear and nonlinear pedotransfer functions were fitted. For soils with soil organic carbon (SOC) greater than 10 g kg^{-1}, the most accurate formula, with an R^2 of 0.45, was used: BD = $1/(0.665+0.00719\times SOC)$. The study area was divided into six agroecological zones (Supplementary Fig. S1) that are widely used in soil carbon pool studies in China [7,8]. The mean values of BD under different land use types were calculated for each zone and were used for soils with SOC less than 10 g kg^{-1}. There were few data on exchangeable calcium (Exch. Ca) in the 1980s and 2010s, most of which were from locations in Hubei, Henan, Sichuan and Guangdong provinces, in the south-central and southwestern zones.

Data analysis

The repeated sampling sites in the 2010s were not perfectly matched to those in the 1980s due to human disturbance (land use change) and the lack of accurate longitudinal and latitudinal information on legacy points. We established the paired contrasts from samples in the 1980s and 2010s, followed by the paired t-test to evaluate their difference. Only samples in cropland, forestland and grassland were considered. First, samples in the 1980s were taken as control plots. Samples in which carbonate was not found were excluded from the analysis. We conducted a spatial analysis of the location in ArcGIS 10.2 (ESRI Inc., USA) to calculate the buffer areas of sites in the 1980s using a given distance (50 km). This step generated a polygon map with several circles whose centers

were the points in the 1980s. Then, the points in the 2010s within these circles were taken as paired points if their pedological information (e.g., land use and parent material) was the same as that of the centered points in the 1980s. The maximum distance between paired points was less than 50 km, and the mean distance between all pairs was 22.6 km. This distance threshold was sufficient to perform the paired t-test analysis at the national scale, as all the paired sites were characterized with the same land management practices. We also calculated the spatial autocorrelation ranges (~300 km) [9], which were much greater than 22.6 km. Finally, a total of 2299 paired samples were grouped in the local area (Fig. 1). For comparison, we also performed paired t-tests based on paired samples between which the maximal distance ranged from 10 km to 50 km (Supplementary Fig. S2).

In addition, changes in SICD in the 1980s, 2000s and 2010s were also assessed by unpaired t-tests due to the different numbers and locations of soil data. The skewed data (Fig. 1) were square-root transformed to a normal distribution prior to the t-test. Since the data on Exch. Ca were few, we used an unpaired t-test to examine whether there was a significant increase in Exch. Ca.

For the pathway analysis (Fig. 2), the information on erosion rates, leaching and accumulation rates, and NH_x and NO_y deposition from 1970 to 2010 was derived from [10], [11] and [12], respectively. According to the Third National Soil Erosion Survey [10], the rate of annual soil erosion was approximately equal to a decrease in soil depth of 0.3~1.0 cm. The overall changes in soil pH for cropland, forest and grassland were obtained from [13], [14] and [15], respectively. The national fertilizer consumption data

between 1980 and 2010 were provided by the National Bureau of Statistics of China (http://www.stats.gov.cn/). The average wind erosion in China significantly declined from 1990 (12.32 t ha^{-1} yr^{-1}) to 2015 (6.60 t ha^{-1} yr^{-1}) [16], most of which occurred in arid and semiarid regions, half of which fell out beyond [17]. Therefore, we recalculated the average wind erosion rates according to the SIC values, soil wind erosion modulus [16] and the proportions of land use in different regions.

Estimation of soil inorganic carbon stock

Widely used digital soil mapping techniques were adopted to model the spatiotemporal pattern of SICD. It was assumed that soil variation can be modeled as a function of soil-forming factors, such as climate and terrain variables [18]. Four algorithms were considered to establish predictive models: geographically weighted regression, random forest (RF), quantile regression forest and extreme gradient boosting. An ensemble prediction was adopted if several techniques achieve similar accuracy (e.g., the difference in R^2 values of two models was less than 5%). In different predictive cases, RF obviously outperformed other algorithms (Supplementary Fig. S6) and was used to generate SICD maps (Supplementary Figs. S5 and S8). The RF algorithm was employed with the R package “randomForest” [19].

The significance of deep soil C should not be neglected. Approximately 47% of SOC is located in subsoils (30-100 cm) [20], and considerable SIC is stored deeper than 1 m [21,22]. The SIC stocks in the topmost meter in China are estimated to be 53.3-77.9 Pg C [2,3,23], which are slightly less than those of SOC, but the SIC stocks of soil

profiles (up to 2-3 m) could be 234.2 Pg C, which are much more than the SOC pool (147.9 Pg C) [2]. Here, we also had interest in the SIC pool in subsoils (>30 cm). Since many soil pits were dug at soil depths less than 2 m, we fitted the depth functions for samples missing values down to 3 m depth. In addition to compiling SIC data from publications published during the 2000s, we synthesized SIC data for which the sampling depths were deeper than 2 m from publications published between 2011 and 2020 (Supplementary Table S7). Soil profiles with sampling depths deeper than 1 m were used to fit the depth functions of SIC mass density as follows:

$$SICMD(dep) = \begin{cases} f_{exp}(dep) = a_1 \times \exp(b_1 \times dep) \\ f_{power}(dep) = a_2 \times dep^{b_2} \\ f_{loga}(dep) = a_3 \times \log(dep) + b_3 \\ f_{poly}(dep) = a_4 \times dep^2 + b_4 \times dep + c_4 \end{cases}, \quad (2)$$

where *SICMD* is the SIC mass density (kg C m^{-3}), *dep* is the depth of the soil sample (m), and a_1, b_1, a_2, b_2, a_3, b_3, a_4, b_4, and c_4 are the parameters of the exponential function (f_{exp}), power function (f_{power}), logarithmic function (f_{loga}) and second-order polynomial function (f_{poly}). The depth functions were separately fitted in each land use of China's zones divided by geography, climate and agroecology [2,3,7]. After a thousand rounds of fitting, the models fitted regarding geographic zones [2] were the most accurate. The optimal-fitted depth functions (Supplementary Fig. S7) were adopted to generate SICD values lacking to a soil depth of 3 m. The mean value of one soil profile was used for subsoil layers if depth function fitting failed. The SICD of each site was aggregated to 0-1 m, 1-2 m and 2-3 m depth intervals.

In general, topsoils are very sensitive to environmental changes, and therefore, many studies on SIC dynamics have been performed on topsoil [1,24]. The depth

function may involve uncertainty (Supplementary Fig. S7). Therefore, topsoil (0-30 cm) SICD maps were generated for the 1980s and 2010s, and SICD at soil depths of 0-1 m, 1-2 m and 2-3 m was interpolated for the 2010s. The total SIC stocks greatly depended on soil depth. Hence, the ensemble means of three published soil depth maps [25-27] were used to mask the produced soil maps. The soil mapping for each case was performed 100 times, in which resampling without replacement was adopted to select 90% of samples for prediction, and the rest was used for validation during each run. The mean values and standard deviations (STDs) of predictions were used to assess the uncertainty in soil mapping. Regarding the large computation requirement, we developed a new package of "ParallelDSM" [28] for parallel computing of soil mapping.

Data-driven future projection

To quantify the long-term SIC changes, trained predictive models were used to perform projections over the 21st century by incorporating dynamic environmental conditions. The predictive model in the 2010s could be extrapolated to future scenarios while holding the model's parameters constant and incorporating covariates between 2020 and 2100 [29,30]. According to the identified spatial controls (Supplementary Figs. S9 and S10), the cumulative effect of future anthropogenic activity (i.e., land use change), climate change and N deposition on SIC dynamics was evaluated.

The environmental variables included climatic variables, N deposition, land use and terrain attributes. Variables that were significantly correlated with SICD ($P < 0.05$) were selected by stepwise regression in both directions. Variables with a variance

inflation factor greater than 5 were removed. The remote sensing variables were not considered because they were unavailable under future scenarios. The Shuttle Radar Topography Mission digital elevation model [31] at 90 m resolution was employed to generate terrain attributes using SAGA GIS (http://saga-gis.org/): elevation, plan curvature, profile curvature, slope, slope position, multiresolution index of valley bottom flatness, topographic wetness index, and terrain ruggedness index.

Three kinds of future dynamic variables that were derived mainly from Coupled Model Intercomparison Project Phase 6 (CMIP6) models were used, including climatic variables (mean annual precipitation (MAP) and mean annual temperature (MAT)), land use (cropland, forest and grassland) and N deposition variables (NH_x and NO_y). Two Shared Socioeconomic Pathway (SSP) scenarios were considered: SSP1-2.6 and SSP3-7.0, representing the low and medium/high radiative forcing, respectively [32]. SSP1-2.6 was a new version of representative concentration pathways (RCP) 2.6 in CMIP5. The climatic variables at 2.5-minute resolution for 2021-2100 were obtained from WorldClim 2.0 (https://worldclim.org) [33]. For each SSP, climatic variables were produced from 9 global climate models (GCMs). Only the MAP and MAT were used to avoid a multicollinearity problem. Global N deposition data were acquired from [34]. This dataset was produced specifically in support of CMIP6 at a resolution of 1.9°×2°. The land use maps at a 1-km resolution for 2010-2100 were obtained from [35]. These land use maps were downscaled from future land use scenarios for 2 RCPs in CMIP5: RCP 2.6 and RCP 6.0, which approximately correspond to SSP1-2.6 and SSP3-7.0, respectively [32]. These maps might be the finest-scale and thus were adopted, as the

future land use maps within CMIP6 models were characterized with coarser resolutions. To produce soil maps at a finer resolution, all the environmental variables were resampled to 250 m. Due to the large computing cost of running the predictive model 100 times, the prediction uncertainty for each future scenario was not assessed. The minimal and maximal projections were presented to provide information on the variation in SIC stocks.

Supplementary Figures

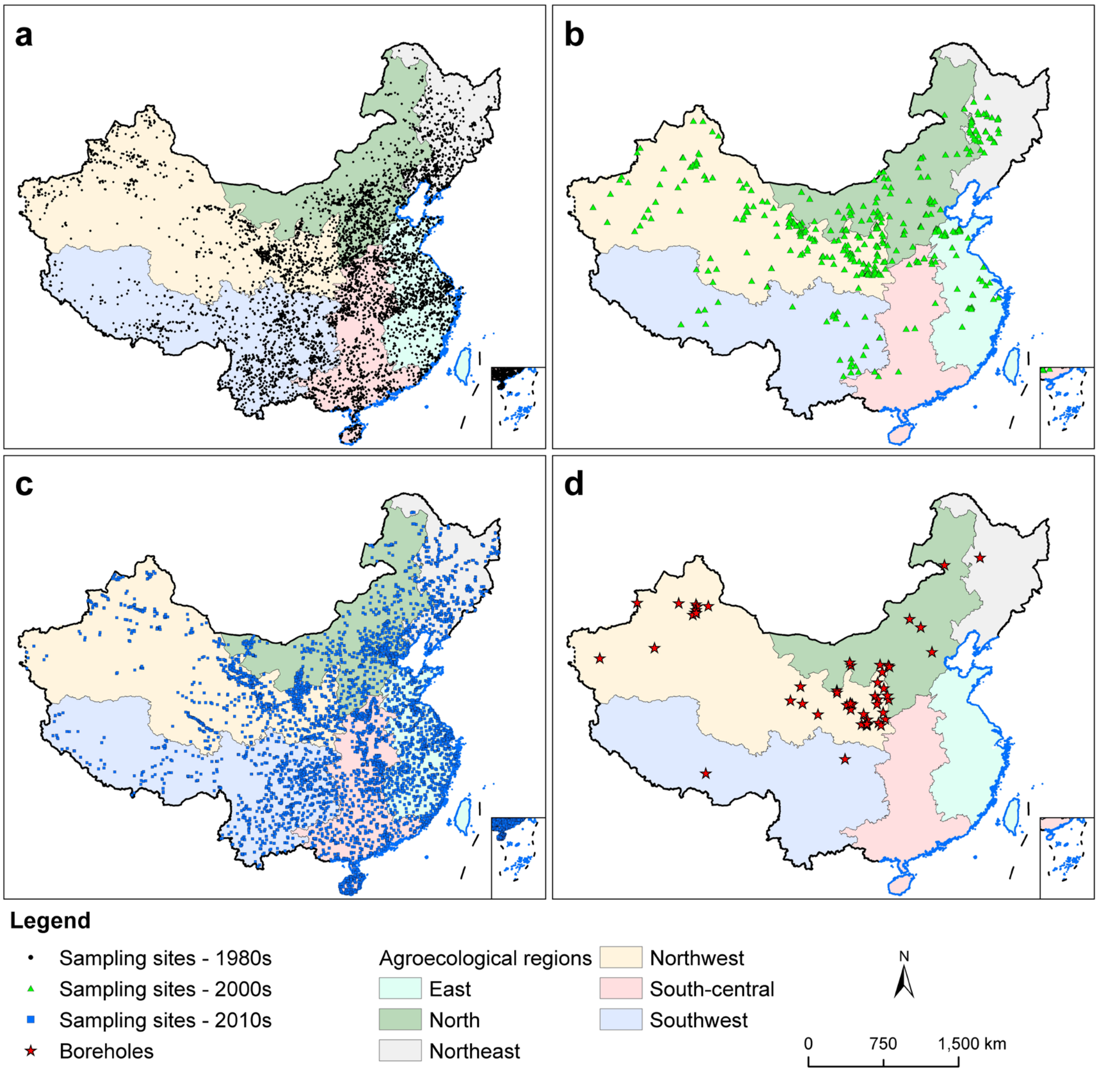

Supplementary Figure S1. Sampling sites over six agroecological zones in different periods. (a) Soil data collected from monographs in the 1980s. (b) Soil data collected from publications in the 2000s. (c) Soil data collected from national soil sampling in the 2010s. (d) Boreholes with sampling depths greater than 2 m, which were synthesized from publications in 2000-2020. Some symbols overlap because their locations are adjacent to each other.

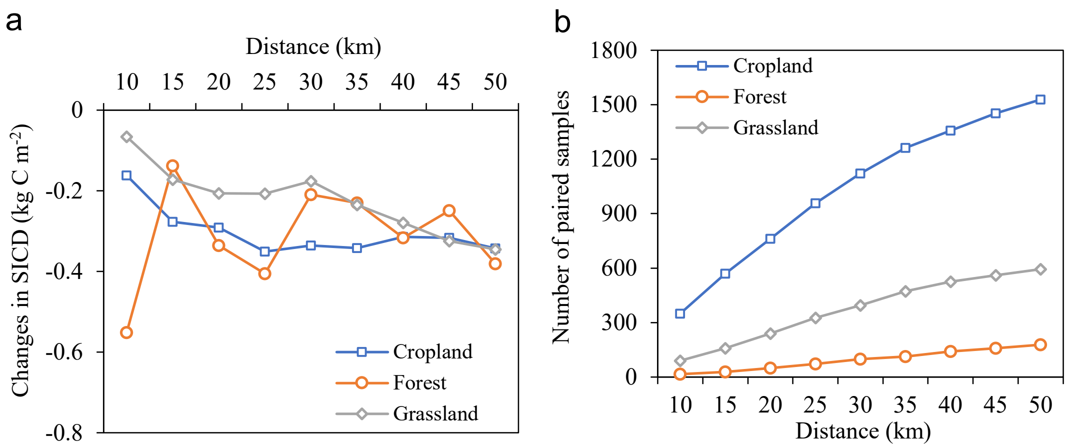

Supplementary Figure S2. Changes in the SICD of paired samples and the number of soil samples when increasing the maximal Euclidean distance between paired samples. (a) Changes in SICD based on the paired t-test. (b) The number of paired samples.

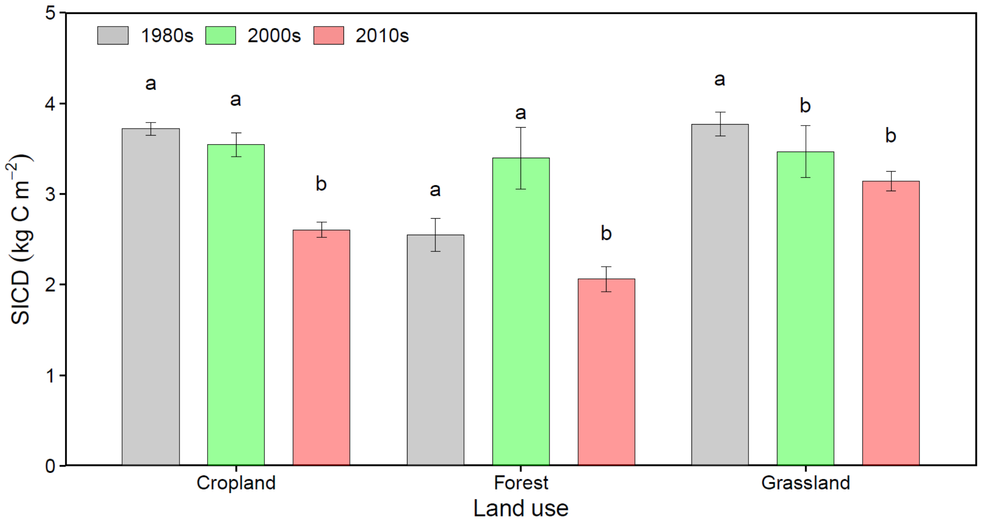

Supplementary Figure S3. Changes in SICD between the 1980s and 2010s. An unpaired t-test was conducted to examine the difference between treatments. The error bar represents the standard error.

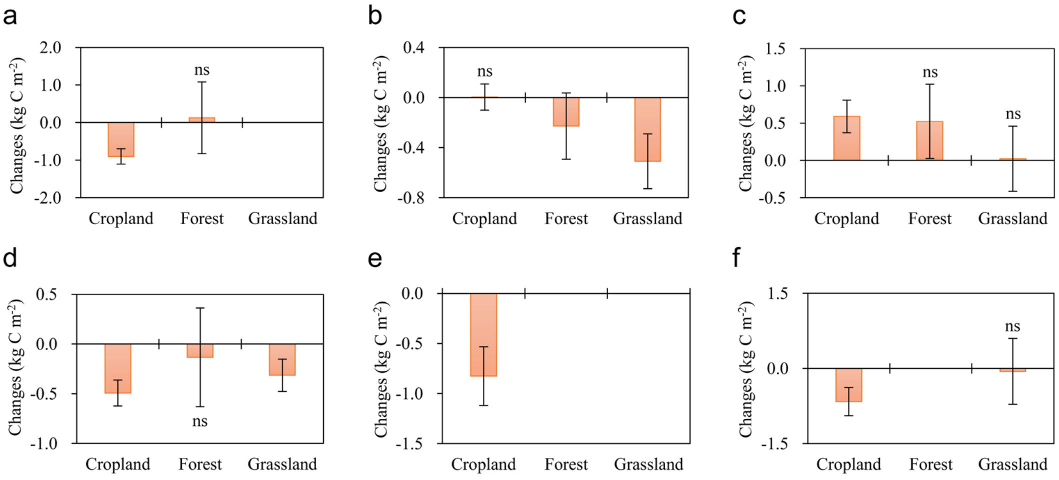

Supplementary Figure S4. Changes in SICD in different agroecological zones. A paired t-test was conducted for topsoil (0-30 cm) in East China (a), North China (b), Northeast China (c), Northwest China (d), South-central China (e) and Southwest China (f). ns indicates that changes are not significant ($P > 0.05$). Due to limited soil data, the changes in SICD in grassland and forest were not analyzed in East, South-central and Southwest China. The error bar represents the standard error.

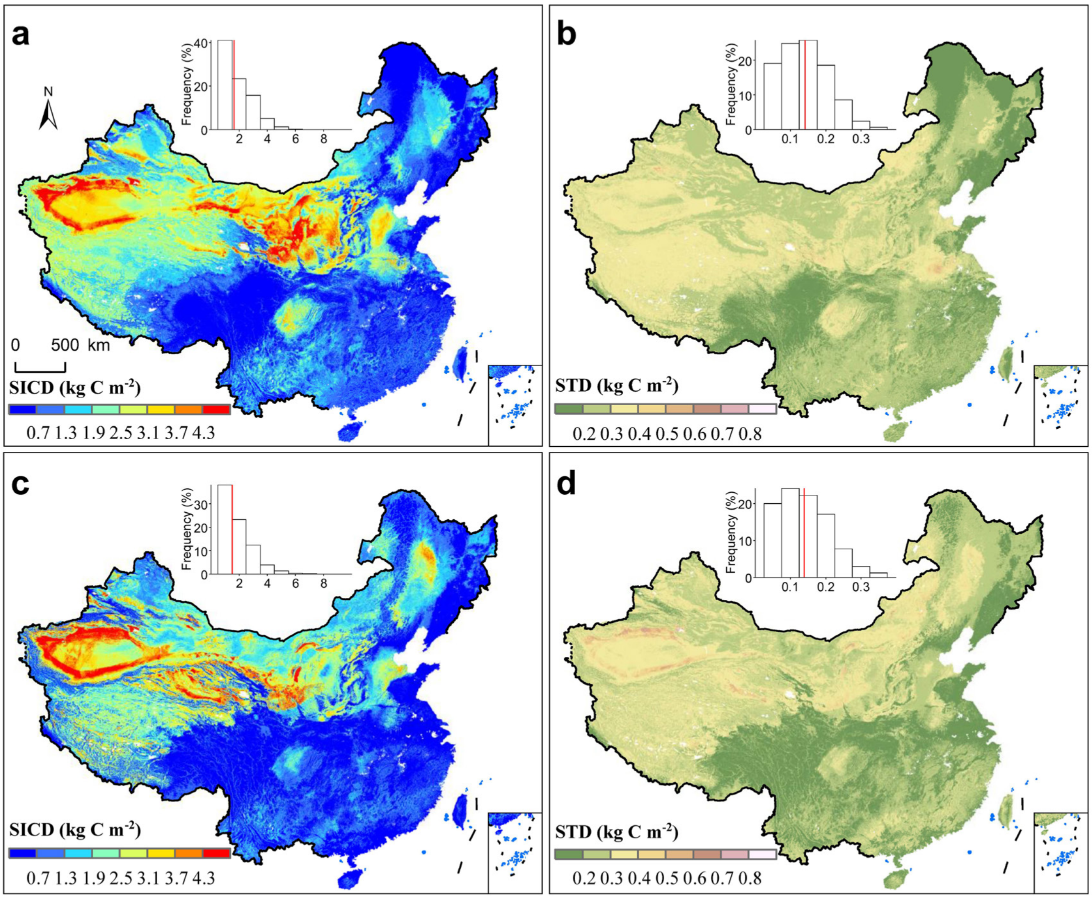

Supplementary Figure S5. Spatial distribution of topsoil (0-30 cm) SICD across China from the 1980s to 2010s. (a) The spatial distribution of the mean SICD values for the 0-30 cm soil depth based on 100 simulations in the 1980s. (b) The spatial distribution of the standard deviation (STD) of predictions based on 100 simulations in the 1980s. (c-d) Same as (a-b) but for the 2010s. The inset is the relative frequency histogram of each map, in which the red line indicates the mean value.

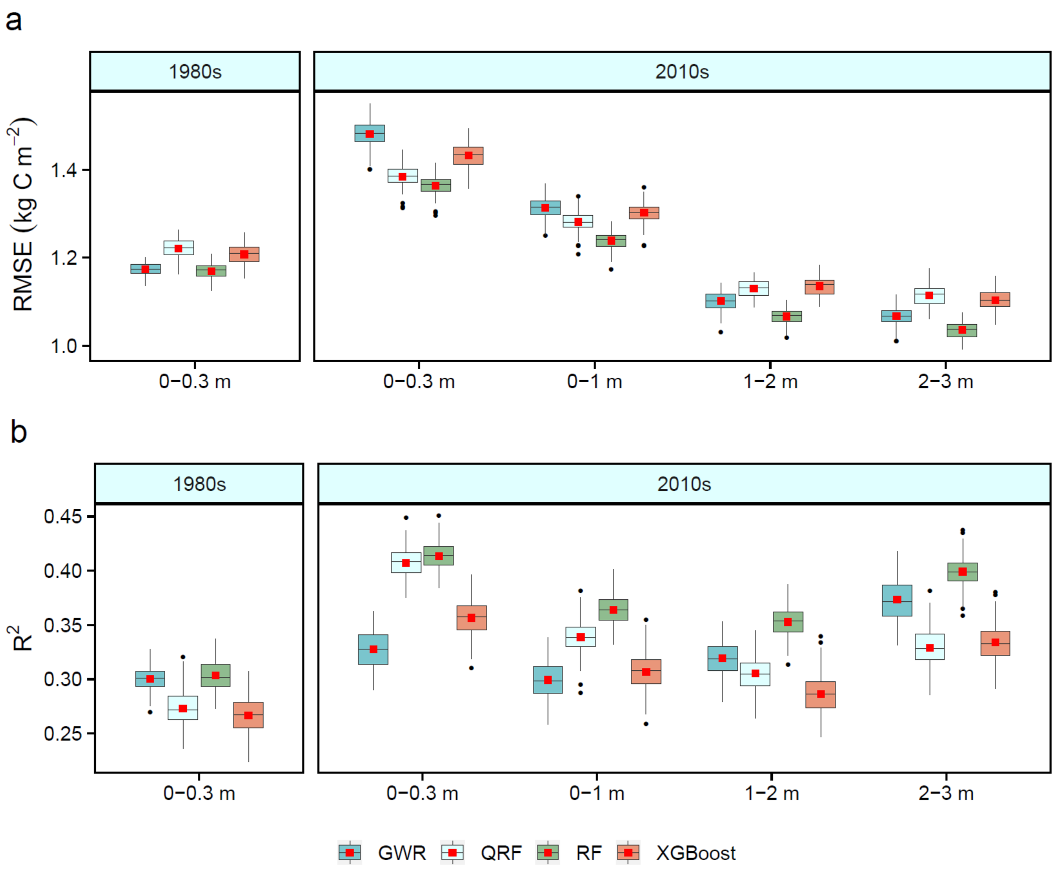

Supplementary Figure S6. Box plots for the accuracy evaluation of 100 predictions. Prediction accuracy was evaluated in terms of the root mean square error (RMSE) (a) and the coefficient of determination (R^2) (b). The red-solid square in each box represents the mean value. GWR: geographically weighted regression, QRF: quantile regression forest, RF: random forest, XGBoost: extreme gradient boosting.

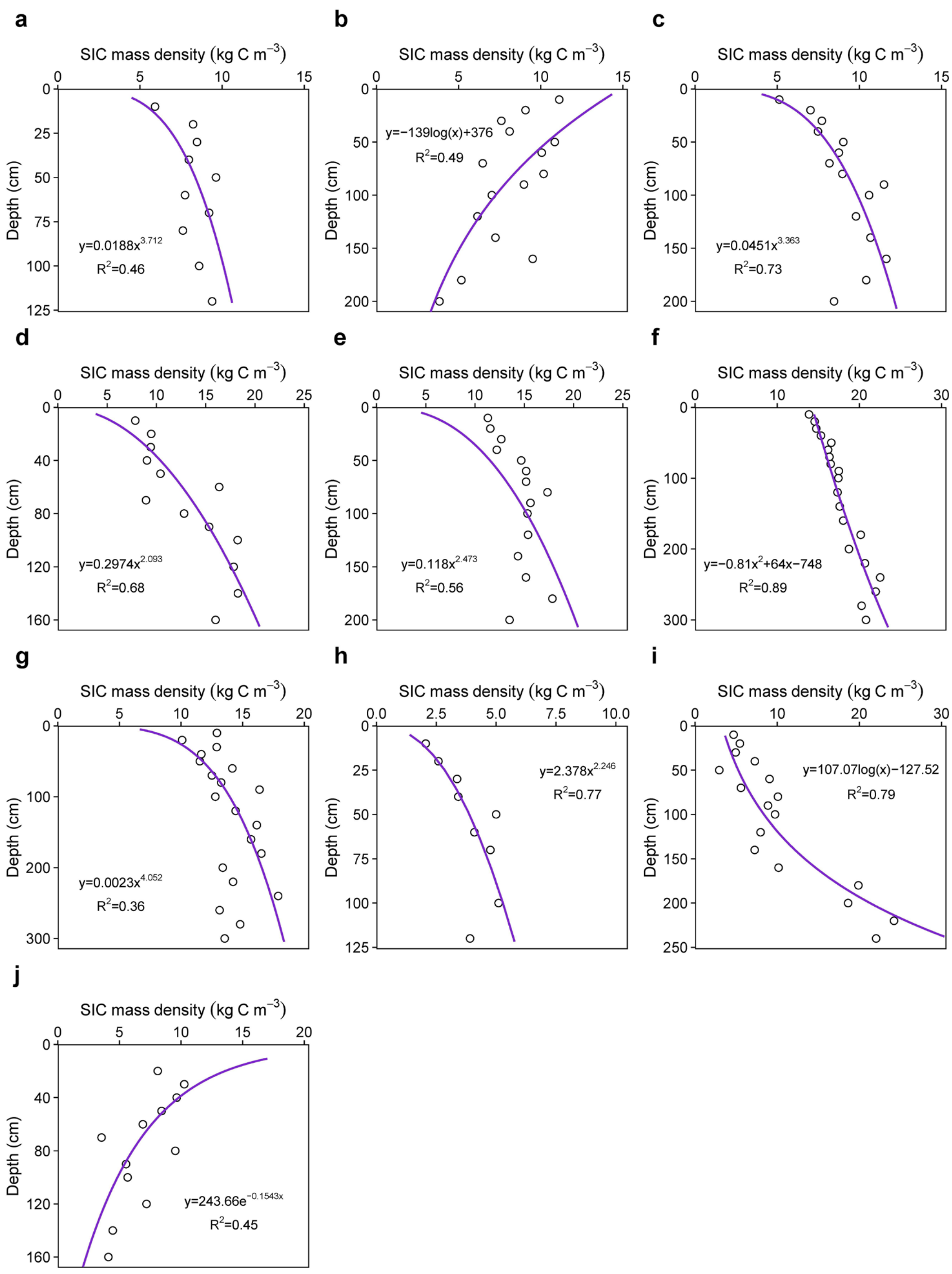

Supplementary Figure S7. Depth functions for SIC mass density fitted based on the observations. Depth functions were successfully fitted for the upland in the eastern zone (a), grassland (b) and upland (c) in the northern zone, grassland in the northeastern zone (d), grassland (e), upland (f) and forest (g) in the northwestern zone, paddy in the south-central zone (h), and grassland (i) and paddy (j) in the southwestern zone. The black circles indicate mean values of SIC mass density at each depth increment (10 cm). The

exhibited formulas are inverse functions. For inaccurate fitting cases, the mean values of observations of one soil profile were used for the subsoil layers. These models were fitted on the basis of geographic zones [2].

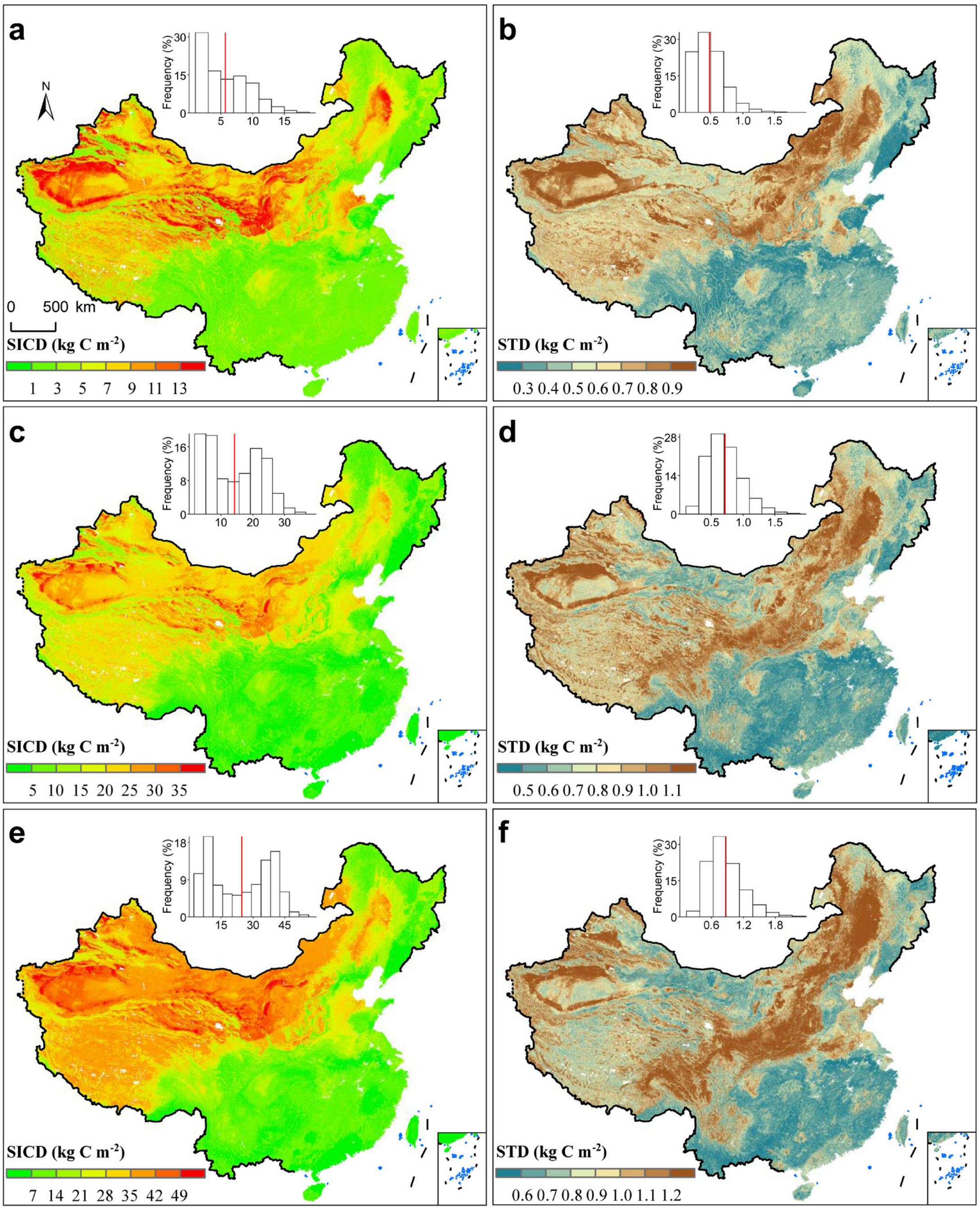

Supplementary Figure S8. Spatial distribution of subsoil SICD in China. (a) The spatial distribution of the mean SICD values for a soil depth of 0-1 m based on 100 simulations. (b) The spatial distribution of the standard deviation (STD) of SICD values for a soil depth of 0-1 m based on 100 simulations. (c-d) and (e-f) Same as (a-b) but for

0-2 m and 0-3 m, respectively. The inset is the relative frequency histogram of each map, in which the red line indicates the mean value.

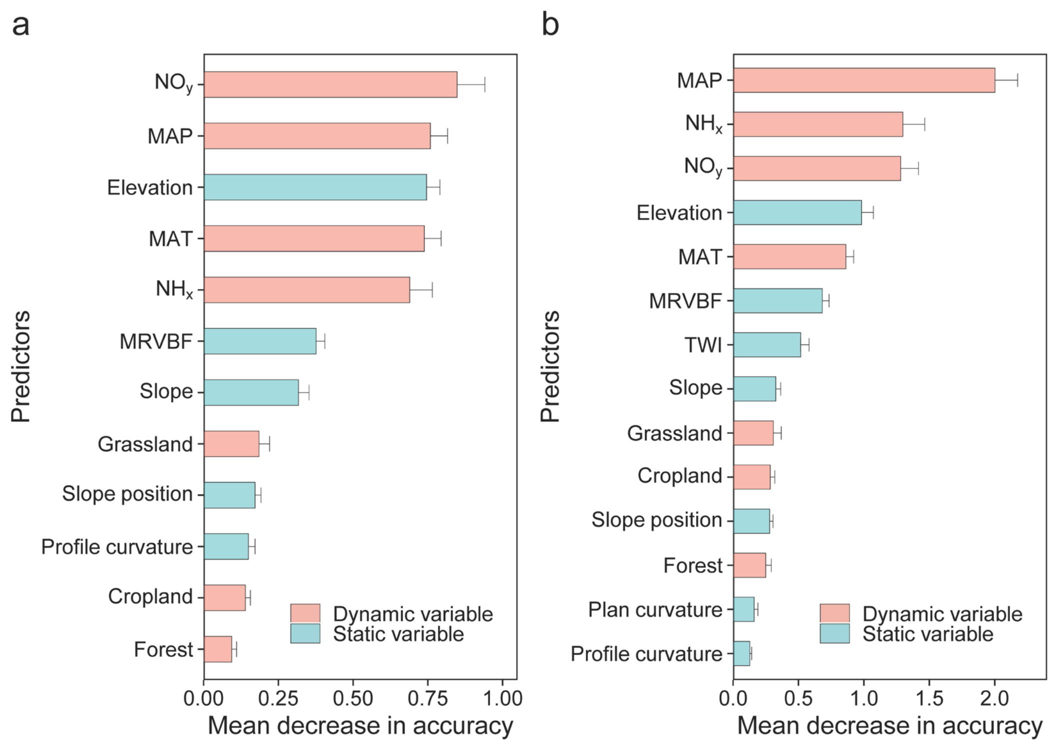

Supplementary Figure S9. The relative importance of environmental variables controlling topsoil SICD. (a) Predictive model established for the 1980s. (b) Predictive model established for the 2010s. The error bars represent standard errors. Variable importance was determined using random forest models in terms of the mean decrease in accuracy (MDA). The greater the MDA is, the more importance this predictor has. Static variables refer to the terrain attributes that generally do not change with time. Predictors are shown on the left: MAP: mean annual precipitation, MAT: mean annual temperature, MRVBF: multiresolution index of valley bottom flatness, NH_x: cumulative wet and dry NH_x deposition over the past decade (1971-1980 for the 1980s and 2001-2010 for the 2010s), NO_y: cumulative wet and dry NO_y deposition, TWI: topographic wetness index. Cropland, forest, and grassland refer to land use.

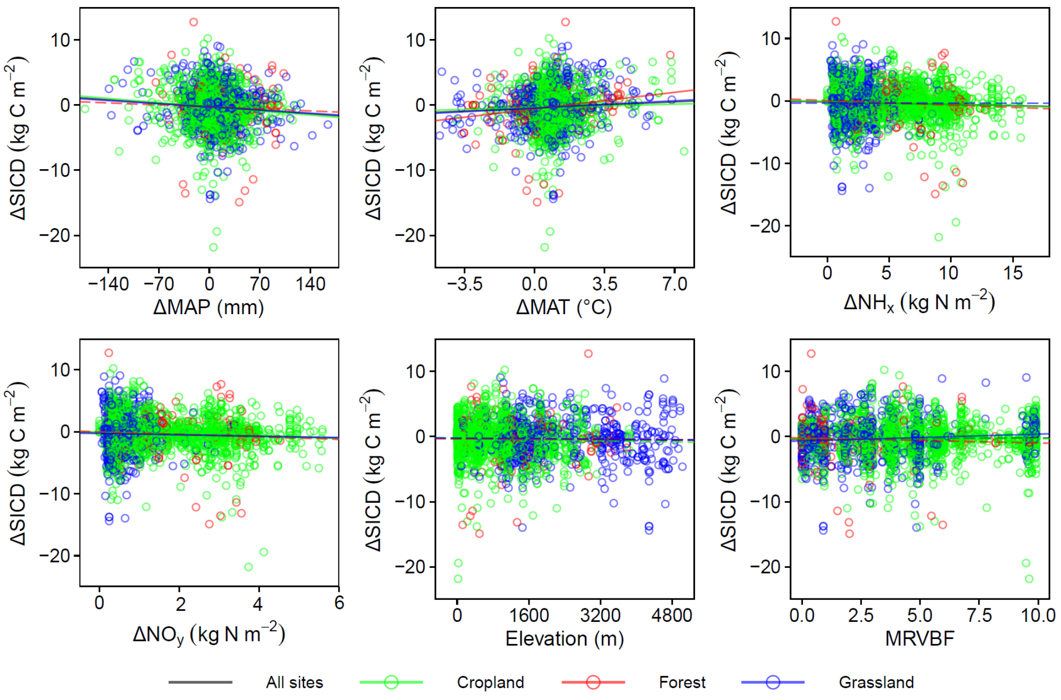

Supplementary Figure S10. Changes in SICD with important environmental variables. Significant fits (P < 0.05) are illustrated by solid lines, and nonsignificant fits are represented by dashed lines. ΔMAP, ΔMAT, ΔNH_x and ΔNO_y are the changes in MAP, MAT, NH_x and NO_y, respectively, between 1981 and 2010.

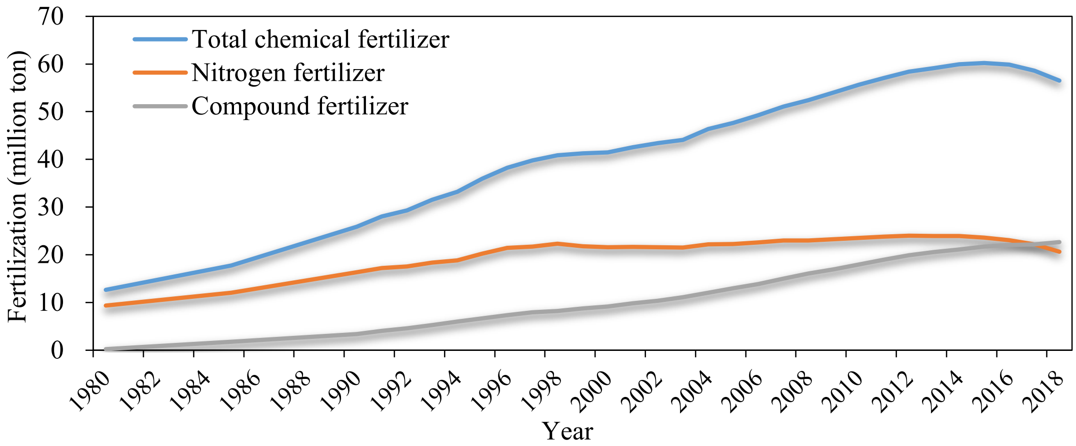

Supplementary Figure S11. Changes in chemical fertilizer consumption in mainland China. Changes in the consumption of total chemical fertilizer, nitrogen fertilizer and compound fertilizer between 1980 and 2018 are illustrated by blue, orange and gray lines, respectively, which were obtained from the National Bureau of Statistics of China (http://www.stats.gov.cn/). Data for 1981-1984 and 1986-1989 and data for the islands of the South China Sea, Macao, Hong Kong and Taiwan are not included.

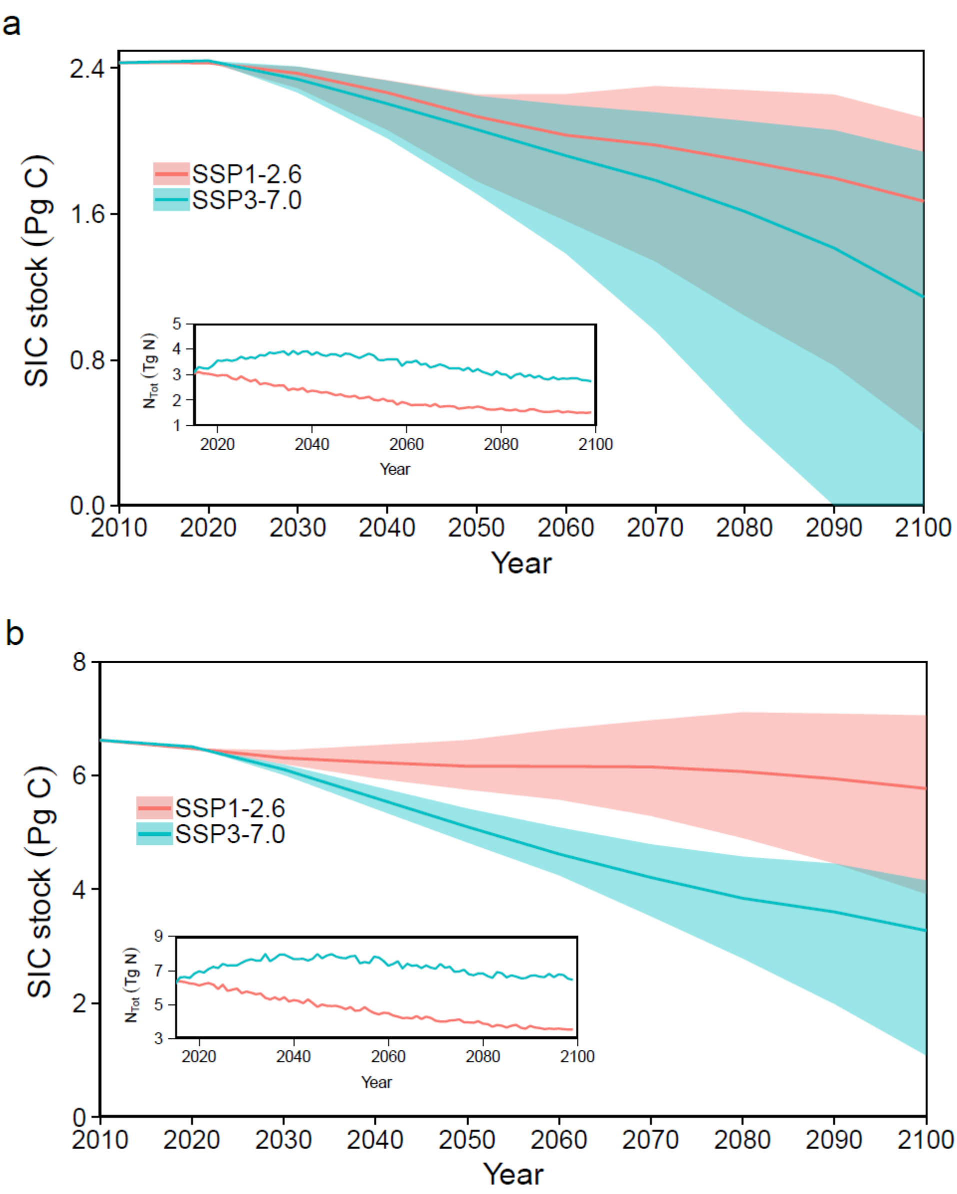

Supplementary Figure S12. Trend in the topsoil SIC stocks in China for the period of 2010-2100 under the SSP1-2.6 and SSP3-7.0 scenarios. (a) China's agroecosystem. (b) China's natural ecosystems, including forest and grassland. The solid lines represent the mean values, and the shaded regions indicate the predicted values from 9 CMIP6 models. The changes in total N deposition (N_{Tot}) are illustrated in insets, which were obtained from [34]. The time step for SIC stocks changes is modeled in the decades from 2010 to 2100, aiming to better quantify the cumulative effect of global change, including land use, climate change and N deposition, on SIC.

Supplementary Tables

Supplementary Table S1. Comparisons with related studies on the SIC stocks in China.

No.	Sources	Study area	Sampling sites	Soil depth	Year	Method	Main conclusions
Estimation of the SIC stock							
1	[36]	Upper Yellow River Delta (NC)	31	1 m	2015	Statistical analysis	• SOC was significantly positive correlated with SIC in cropland (r=0.88, $P < 0.001$).
2	[2]	China	2,456	1 m	1980s	Soil type linkage with expansion factors for deep soil carbon	• The SOC stock and SIC stock at a soil depth of 1 m were 83.8 Pg and 77.9 Pg C, respectively.<br>• For the entire soil profile, the SOC stock and SIC stock were 147.9 Pg and 234.2 Pg C, respectively.
3	[23]	China	2,473	1 m	1980s	Soil type linkage method	• The SIC stock at a soil depth of 2 m in China was approximately 53.3±6.3 Pg C.
4	[37]	Loess Plateau (approximately NW)	495	1 m	1980s	Soil type linkage method	• The SIC stock (1 m) was approximately 10.20 Pg C.
5	[38]	Inner Mongolia (NC)	15	3 m	-	Statistical analysis	• At least 50% of soil C was stored in the 1-3 m soil layer, of which the main contributor was SIC for shrub desert and desert.
Decrease in the SIC stock							
6	[39]	Fuxian County (NW)	10	1 m	2009	Statistical analysis	• 30-year afforestation resulted in a decline in the topsoil (20 cm) SIC stock.

No.	Sources	Study area	Sampling sites	Soil depth	Year	Method	Main conclusions
7	[40]	Loess Plateau (approximately NW)	84	3 m	2016	Statistical analysis	• Vegetation restoration resulted in a decrease in the SIC stock.<br>• For the upper 3 m of soil, at least 50% of C was stored at depths of 1-3 m.
8	[41]	China	1,635	1 m	1980s	Statistical analysis	• Soil acidification decreased the SIC but increased the SOC in carbonate soils.
9	[42]	Gangcha County (NW)	36	0.3 m	2012	Statistical analysis	• Ten-year restoration resulted in an increased SOC stock and a decreased SIC stock in grassland.
10	[43]	Yunwu Mountain (NW)	9	2 m	2011	Isotopes method	• 22-year grassland restoration resulted in a decrease in the SIC stock due to soil acidification.
11	[44]	Changwu County (NW)	15	1 m	2012	Statistical analysis	• 30-year grassland restoration led to a decrease in the SIC stock (75 g m^{-2} y^{-1}).
12	[1]	China	34,411	1 m	1980s	Soil type linkage	• N-induced soil acidification led to significant loss of SIC stocks in Chinese croplands.<br>• Approximately 0.15 Pg SIC (40 cm) was lost from 1980 to 2020.
13	[45]	Inner Mongolia and Tibetan Plateau (NC, NW and SW)	81	20 cm	2003-2007	Machine learning and statistical analysis	• SIC was controlled by soil acidification and other processes associated with soil pH.<br>• Soil acidification may result in a 53% and 30% decrease in SIC in the Tibetan grasslands and Inner Mongolian grasslands, respectively.
14	[22]	Loess Plateau (approximately NW)	12	5 m	2014	Statistical analysis	• Afforestation decreased SIC at high SICs site but increased SIC at low SIC sites.
15	[46]	Ziwuling Mountains (NW)	18	1 m	2013	Statistical analysis	• The topsoil SIC stock decreased with restoration age.

No.	Sources	Study area	Sampling sites	Soil depth	Year	Method	Main conclusions
16	[3]	China	2,553	1 m	1980s	Soil type linkage method	• The SIC stock at a soil depth of 1 m in China was approximately 55.3±10.7 Pg C.<br>• Half of the cultivated land lost topsoil SIC.<br>• A total of 1.6 Pg of SIC was lost.
17	[24]	Tibetan Plateau (NC, SW and NW)	602	10 cm	2001-2005	Geo-statistics	• Topsoil (10 cm) SIC stocks decreased significantly (26.8 g C m^{-2} yr^{-1}) in grassland.<br>• Great SIC loss was generally found in areas with strong soil acidification.
18	[47]	Qingyang City (NW)	28	1 m	2013	Isotopes method	• After a 60-year vegetation restoration period, grasslands stored less SIC than that of forestland but generated more pedogenic carbonate.
Increase in the SIC stock							
19	[48]	Hebei Province (NC)	5	1.6 m	2013	Isotopes method	• Fertilization significantly increased pedogenic carbonate by the neoformation of pedo-atmogenic carbonates.
20	[49]	Yanchi Research Station (NW)	63	1 m	-	Isotopes method	• Vegetation rehabilitation on sand land sequestered SIC and SOC in semiarid deserts.
21	[50]	Yanchi County (NW)	13	1 m	2015	Isotopes method	• Afforestation sequestered SIC in terms of pedogenic carbonate.
22	[51]	Hebei Plain (NC)	24	1 m	2015	Statistical analysis	• SIC was strongly positively correlated with SOC (r=0.74).<br>• SIC stock (1 m) was twice more than SOC stock.
23	[52]	Linze County (NW)	21	15 cm	2007	Statistical analysis	• Rehabilitation of severe sandy desertified land enhanced the SOC and SIC concentrations.
24	[53]	Yanqi Basin (NW)	21	1 m	2010	Isotopes method	• Cultivation enhanced the pedogenic carbonate.

No.	Sources	Study area	Sampling sites	Soil depth	Year	Method	Main conclusions
25	[54]	Yanqi Basin (NW)	26	1 m	1996, 2002, 2009 and 2010	Statistical analysis	• SIC may account for more than 80% of the total carbon in arid regions.<br>• Converting shrub land to cropland led to an increase in pedogenic carbonate.<br>• SOC had a significant relationship with SIC.
26	[55]	Songnen Plain (NE)	5	1 m	2011	Statistical analysis	• For the upper 1 m of soil, cultivation (over 24 years) led to a decrease in SOC (363 g C m^{-2} yr^{-1}) and an increase in SIC (53 g C m^{-2} yr^{-1}).
27	[56]	Lanzhou City (NW)	33	2 m	2012	Statistical analysis	• Irrigation increased the topsoil (0-30 cm) SIC stock by 10%.<br>• The subsoil (1-2 m) SOC and SIC stocks were approximately 40% and 50% of the total stocks in the 0-2 m soil layer, respectively.

Supplementary Table S2. Total SIC stocks (0-30 cm) regarding land use and agroecological zones. The values are presented as the means ± standard deviations.

Zone	1980s (Pg C)	2010s (Pg C)	Changes (%)	Zone	1980s (Pg C)	2010s (Pg C)	Changes (%)
East	0.76±0.02	0.58±0.02	-23.68±3.56	*Northwest*	7.22±0.14	6.76±0.11	-6.37±2.58
Cropland	0.38±0.01	0.26±0.01	-31.58±2.73	Cropland	0.75±0.01	0.67±0.01	-10.67±1.33
Forest	0.24±0.01	0.22±0.02	-8.33±7.17	Forest	0.22±0.01	0.19±0.01	-13.64±2.87
Grassland	0.04±0.01	0.03±0.01	-25.00±4.92	Grassland	2.25±0.05	2.22±0.03	-1.33±3.01
North	2.81±0.05	2.56±0.06	-8.90±2.76	*South-central*	1.02±0.04	0.75±0.03	-26.47±4.03
Cropland	0.57±0.01	0.54±0.01	-5.26±2.12	Cropland	0.39±0.02	0.27±0.01	-30.77±3.22
Forest	0.20±0.01	0.23±0.01	15.00±5.84	Forest	0.50±0.02	0.39±0.02	-22.00±5.67
Grassland	0.97±0.03	0.97±0.04	0±4.96	Grassland	0.05±0.01	0.04±0.01	-20.00±4.09
Northeast	0.72±0.02	0.81±0.03	12.50±4.71	*Southwest*	2.93±0.11	2.61±0.07	-10.92±3.88
Cropland	0.37±0.01	0.40±0.01	8.11±4.74	Cropland	0.45±0.01	0.29±0.01	-35.56±2.27
Forest	0.20±0.01	0.20±0.01	0±8.15	Forest	0.54±0.02	0.44±0.01	-18.52±3.10
Grassland	0.05±0.01	0.07±0.01	40.00±6.03	Grassland	1.61±0.08	1.62±0.06	0.62±5.86
Total [1)]	15.46±0.27	14.07±0.20	-8.99±2.24				

[1)] The total SIC stock in six agroecological zones.

Supplementary Table S3. Total SIC stocks (0-3 m) regarding land use and agroecological zones. The values are presented as the means ± standard deviations.

Zone	0-1 m (Pg C)	0-2m (Pg C)	0-3 m (Pg C)	Zone	0-1 m (Pg C)	0-2m (Pg C)	0-3 m (Pg C)
East	2.28±0.06	5.46±0.10	8.75±0.12	*Northwest*	24.82±0.37	62.52±0.54	108.57±0.62
Cropland	1.14±0.03	2.88±0.05	4.71±0.05	Cropland	2.42±0.03	5.62±0.04	9.23±0.05
Forest	0.75±0.04	1.59±0.06	2.41±0.07	Forest	0.78±0.01	2.15±0.04	3.91±0.05
Grassland	0.10±0.01	0.24±0.01	0.39±0.01	Grassland	8.69±0.11	21.88±0.20	38.04±0.23
North	10.64±0.21	27.35±0.26	46.97±0.33	*South-central*	2.72±0.09	6.04±0.14	9.47±0.18
Cropland	2.11±0.04	5.04±0.05	8.40±0.07	Cropland	1.05±0.03	2.42±0.04	3.86±0.06
Forest	1.06±0.04	2.65±0.08	4.66±0.10	Forest	1.30±0.06	2.79±0.09	4.28±0.11
Grassland	4.07±0.13	10.95±0.14	18.94±0.16	Grassland	0.14±0.01	0.30±0.01	0.48±0.01
Northeast	3.33±0.09	7.28±0.19	11.46±0.25	*Southwest*	9.79±0.23	26.31±0.38	46.99±0.48
Cropland	1.63±0.03	3.62±0.07	5.68±0.10	Cropland	0.95±0.03	1.98±0.04	3.02±0.05
Forest	0.85±0.05	1.83±0.11	2.96±0.13	Forest	1.52±0.05	3.69±0.09	6.23±0.13
Grassland	0.29±0.01	0.61±0.01	0.94±0.02	Grassland	6.28±0.16	17.47±0.26	31.61±0.30
Total [1)]	53.58±0.57	134.96±0.91	232.21±1.21				

[1)] The total SIC stock in six agroecological zones.

Supplementary Table S4. The changes in exchangeable calcium (cmol (+) kg^{-1}) in China.

Land use	1980s		2010s		Change	*P*
	Exch. Ca	*N* [1)]	Exch. Ca	*N*		
Cropland	6.89	77	9.01	214	2.12	0.029
Forest	3.77	71	5.49	140	1.72	0.026

[1)] *N* refers to the number of samples. Note that only data from Hubei, Henan, Sichuan and Guangdong provinces, which were located in the south-central and southwestern zones (Fig. S1), were used. Due to limited soil samples (*N*<20), the analysis was not performed for grassland.

Supplementary Table S5. Data sources for the SIC data from the 1980s.

No.	Language	Type	Reference
1	Chinese	Monograph	Department of Agriculture of Xinjiang Uygur Autonomous Region. *Soil Species of Xinjiang Uygur Autonomous Region*. Urumchi: Xinjiang Science and Technology Medical Press, 1993.
2	Chinese	Monograph	Ding DZ. *Soil Species of Hebei Province*. Shijiazhuang: Hebei Science & Technology Press, 1992.
3	Chinese	Monograph	Institute of Forestry Soil Science Chinese Academy of Sciences. *Soil of Northeast China*. Beijing: Science Press, 1980.
4	Chinese	Monograph	Land Management Bureau of Tibet Autonomous Region. *Soil Source of Tibet Autonomous Region*. Beijing: Science Press, 1994.
5	Chinese	Monograph	National Soil Survey Office. *Soil Species of China, vol. 1*. Beijing: China Agriculture Press, 1993.
6	Chinese	Monograph	National Soil Survey Office. *Soil Species of China, vol. 2*. Beijing: China Agriculture Press, 1994.
7	Chinese	Monograph	National Soil Survey Office. *Soil Species of China, vol. 3*. Beijing: China Agriculture Press, 1994.
8	Chinese	Monograph	National Soil Survey Office. *Soil Species of China, vol. 4*. Beijing: China Agriculture Press, 1995.
9	Chinese	Monograph	National Soil Survey Office. *Soil Species of China, vol. 5*. Beijing: China Agriculture Press, 1995.
10	Chinese	Monograph	National Soil Survey Office. *Soil Species of China, vol. 6*. Beijing: China Agriculture Press, 1996.
11	Chinese	Monograph	Ningxia Agricultural Engineering Research and Design Institute. *Ningxia soil*. Yinchuan: Ningxia People's Publishing House, 1990.
12	Chinese	Monograph	Qinghai Agricultural Resources and Regional Planning Office. *Soil Species of Qinghai Province*. Beijing: China Agriculture Press, 1995.
13	Chinese	Monograph	Sichuan Agricultural and Pastoral Office and Soil Survey Office of Sichuan Province. *Soil Species of Sichuan Province*. Chengdu: Sichuan Science and Technology Press, 1994.
14	Chinese	Monograph	Soil and Fertilizer Station of Guangxi Zhuang Autonomous Region. *Soil Species of Guangxi Zhuang Autonomous Region*. Nanning: Guangxi Science & Technology Publishing House, 1993.
15	Chinese	Monograph	Soil and Fertilizer Station of Henan Province and Soil Survey Office of Henan Province. *Soil Species of Henan Province*. Beijing: China Agriculture Press, 1995.
16	Chinese	Monograph	Soil and Fertilizer Station of Hubei Province and Soil Survey Office of Hubei Province. *Soil Species of Hubei Province*. Wuhan: Hubei Science & Technology Press, 2015.
17	Chinese	Monograph	Soil and Fertilizer Station of Jilin Province. *Jilin Soil*. Beijing: China Agriculture Press, 1998.
18	Chinese	Monograph	Soil and Fertilizer Station of Liaoning Province. *Soil Species of Liaoning Province*. Shenyang: Liaoning University Publishing House, 1991.
19	Chinese	Monograph	Soil and Fertilizer Station of Shandong Province. *Soil Species of Shandong Province*. Beijing: China Agriculture Press, 1993.
20	Chinese	Monograph	Soil Survey Office of Anhui Province. *Soil Species of Anhui Province*. Beijing: Science Press, 1990.

No.	Language	Type	Reference
21	Chinese	Monograph	Soil Survey Office of Fujian Province. *Fujian Soil*. Fuzhou: Fujian Science & Technology Publishing House, 1991.
22	Chinese	Monograph	Soil Survey Office of Gansu Province. *Soil Species of Gansu Province*. Lanzhou: Gansu Science and Technology Press, 1993.
23	Chinese	Monograph	Soil Survey Office of Guangdong Province. *Soil Species of Guangdong Province*. Beijing: Science Press, 1996.
24	Chinese	Monograph	Soil Survey Office of Guizhou Province. *Soil Species of Guizhou Province*. Guiyang: Guizhou Science and Technology Publishing House, 1994.
25	Chinese	Monograph	Soil Survey Office of Inner Mongolia Autonomous Region. *Soil Species of Inner Mongolia Autonomous Region*. Beijing: China Agriculture Press, 1994.
26	Chinese	Monograph	Soil Survey Office of Jiangsu Province and Phoenix Science Press. *Soil Species of Jiangsu Province*. Nanjing: Phoenix Science Press, 1996.
27	Chinese	Monograph	Soil Survey Office of Shanghai City. *Shanghai Soil*. Shanghai: Shanghai Scientific & Technical Publishers, 1992.
28	Chinese	Monograph	Soil Survey Office of Shanxi Province and Soil Station of Shanxi Province. *Soil Species of Shanxi Province*. Taiyuan: Shanxi Science & Technology Publishing House, 1992.
29	Chinese	Monograph	Soil Survey Office of Yunnan Province. *Soil Species of Yunnan Province*. Kunming: Yunnan Science and Technology Press, 1994.
30	Chinese	Monograph	Soil Survey Office of Zhejiang Province. *Soil Species of Zhejiang Province*. Hangzhou: Zhejiang Science and Technology Press, 1993.

Supplementary Table S6. Data sources for the SIC data from the 2000s.

No.	Language	Type	Reference
1	Chinese	Journal	An S, Chang Q and Li B *et al.* Study on the pedogenesis characteristics and soil taxonomy in the wind-water erosion crisscross zone of north of Shaanxi. *Journal of Northwest Sci-Tech University of Agriculture and Forestry (Natural Science Edition)* 2002; **30**: 27-32.
2	Chinese	Journal	An S, Guo M and Yang J *et al.* Soil Characteristics and Taxonomic Classification in Yunwu Mountain Natural Conservation Area. *Chinese Journal of Soil Science* 2006; **37**: 2209-13.
3	Chinese	Journal	An S, Huang Y and Li B *et al.* Characteristics of soil water stable aggregates and relationship with soil properties during vegetation rehabilitation in a Loess Hilly Region. *Chinese Journal of Soil Science* 2006; **37**: 45-50.
4	Chinese	Dissertation	Bao Y. Study on soil physical and chemical properties of dam land and terrace in the Loess hilly region. *MSc Thesis*. Northwest A＆F University, 2005.
5	English	Journal	Cao J, Zhu C and Chow JC *et al.* Stable carbon and oxygen isotopic composition of carbonate in fugitive dust in the Chinese Loess Plateau. *Atmospheric Environment* 2008; **42**: 9118-22.
6	Chinese	Journal	Chang Q, An S and Liu J *et al.* Study on the characteristic of Land Desertification in the agriculture and animal husbandry interlace zone of northern Shaanxi. *Acta Pedologica Sinica* 2003; **40**: 518-23.
7	English	Journal	Chen B, Kitagawa H and Jie D *et al.* Dust transport from northeastern China inferred from carbon isotopes of atmospheric dust carbonate. *Atmospheric Environment* 2008; **42**: 4790-6.
8	Chinese	Journal	Chen L and Zhang G. Parent material uniformity and evolution of soil characteristics of a paddy soil chronosequence derived from marine sediments. *Acta Pedologica Sinica* 2009; **46**: 753-63.
9	Chinese	Journal	Chen Y and Qin J. Effect of ephedra on soil fertility in gray-brown desert of Hexi Corridor and economic benefits. *Chinese Journal of Soil Science* 2006; **37**: 203-5.
10	English	Journal	Chen Y, Song M and Dong M. Soil properties along a hillslope modified by wind erosion in the Ordos Plateau (semi-arid China). *Geoderma* 2002; **106**: 331-40.
11	Chinese	Journal	Chen Y, Wang J and Zhao W *et al.* The effect of calcium carbonate on sediment aggregation in the intertidal zone of the Yellow River Estuary. *Marine Geology & Quaternary Geology* 2010; **30**: 87-94.
12	Chinese	Journal	Chen Z, Zhang Q and Li X. Enhanced efficacy of phosphate fertilizer mixed with modified-montmorillonite applied in calcareous soil in the Northeast China. *Journal of Agro-Environment Science* 2005; **24**: 158-60.
13	English	Journal	Chun S, Nishiyama M and Matsmoto S. Response of corn growth in salt-affected soils of Northeast China to flue-gas desulfurization by-product. *Communications in Soil Science and Plant Analysis* 2007; **38**: 813-25.
14	Chinese	Journal	Ci E, Gao M and Yu Q. Diagnostic characteristics and taxonomic classification of some main soils along Huaihe River Region in Anhui Province. *Chinese Journal of Soil Science* 2005; **36**: 19-22.
15	Chinese	Journal	Cui H, Wang M and Jie X *et al.* Effect of phosphorus, zinc and cadmium interaction on their availability in calcareous cinnamon soil. *Journal of Agro-Environment Science* 2010; **29**: 97-103.
16	Chinese	Journal	Ding G, Pei W and Li C *et al.* Soil properties and water conservation function of main vegetation types in Dongshan Mountain protection station. *Gansu Science and Technology* 2010; **26**: 163-5.

No.	Language	Type	Reference
17	Chinese	Dissertation	Du H. Correlations between soil organic carbon fractions with variable links to soil physical properties in an alpine pastureland. *MSc Thesis*. Gansu Agricultural University, 2007.
18	Chinese	Journal	Duan W. Suitable types of soils for main tree species of shelter belts on pasture land. *Chinese Journal of Soil Science* 2005; **36**: 57-62.
19	Chinese	Journal	Fan W, Xue X and Yang L *et al.* Effect of cobalt on yield and grain quality of winter wheat. *Plant Nutrition and Fertilizer Science* 2004; **10**: 429-32.
20	Chinese	Journal	Fan W, Yang L and Xue X *et al.* Effect of cobalt application on yield and protein content of winter wheat and available cobalt content in soils. *Chinese Journal of Soil Science* 2005; **36**: 92-5.
21	Chinese	Journal	Fang Z, Zuo Y and Li L *et al.* Effects of different nitrogen levels on iron nutrition and nitrogen fixation of peanut in maize-peanut mixed cropping system. *Plant Nutrition and Fertilizer Science* 2004; **10**: 386-90.
22	Chinese	Journal	Gao L, Shi Y and Yang S. Study on resistance to iron deficiency in peanut cultivars and physiological traits. *Plant Nutrition and Fertilizer Science* 2003; **9**: 480-3.
23	Chinese	Journal	Gao X, Tian Z and Hao X *et al.* The changes of alpine grassland soil nutrient at different deteriorate degree on high mountain meadow of Three River Source. *Journal of Qinghai University (Nature Science)* 2006; **24**: 37-40.
24	Chinese	Journal	Geng Y, Luo G and Yuan G *et al.* Effects of cultivating and grazing on soil organic carbon and soil inorganic carbon in temperate semiarid grassland. *Journal of Agro-Environment Science* 2008; **27**: 2518-23.
25	Chinese	Journal	Gong A, He Y and Huang C *et al.* The classification of typical soil series based on soil taxonomy in Chengdu Plain. *Southwest China Journal of Agricultural Sciences* 2002; **15**: 70-3.
26	Chinese	Journal	Gu Y and Liu F. Taxonomic classification of soils derived from purple-red sand rocks in South Anhui Province. *Acta Pedologica Sinica* 2007; **44**: 776-83.
27	Chinese	Journal	Guan X, Zhang F and Li Q *et al.* Substrate classification of irrigated brown desert soil in south Xinjiang. *Soils* 2003; **1**: 53-7.
28	Chinese	Journal	Guan X, Zhong J and Zhang F. The attribution of "brown desert soil" in soil system classification in Southern Xinjiang. *Soils* 2001; **6**: 289-94.
29	Chinese	Journal	Guo Q, Guo T and Wang Y *et al.* Effect of three sodic salinity on salinization characteristic in limy soils. *Acta Agriculturae Boreali-occidentalis Sinica* 2009; **18**: 155-9.
30	Chinese	Journal	Guo Y, Kato M and Song F *et al.* Composition of loess aggregate and its relationship with CaCO3 on the Loess Plateau. *Acta Pedologica Sinica* 2004; **41**: 362-8.
31	Chinese	Journal	Guo Y, Wang S and Zhang Y *et al.* Relationship of CaCO3 and soil aggregates in irrigated soil of the Loess Plateau, China. *Journal of Shenzhen University Science and Engineering* 2008; **25**: 314-9.
32	English	Journal	He L and Tang Y. Soil development along primary succession sequences on moraines of Hailuogou Glacier, Gongga Mountain, Sichuan, China. *Catena* 2008; **72**: 259-69.
33	Chinese	Journal	He X, Chang Q and Li R *et al.* Study on the soil quality in agriculture and animal interlace zone under the different artificial vegetations. *Research of Soil and Water Conservation* 2005; **12**: 10-2.

No.	Language	Type	Reference
34	Chinese	Journal	He X, Yin L and Yan C *et al.* Genetic characteristics and taxonomy of highland soil in the north front of the Central Tianshan Mountains. *Chinese Journal of Soil Science* 2006; **37**: 833-6.
35	Chinese	Journal	He Y, Huang C and Chen X *et al.* Taxonomic classification of soils derived from loesses in hilly region of Western Sichuan. *Journal of Mountain Science* 2001; **19**: 334-8.
36	English	Journal	Huang B and Gong Z. Geochemical barriers and element retention in soils in different landscapes of the Tianshan Mountain area, Xinjiang, China. *Geoderma* 2004; **126**: 337-51.
37	Chinese	Journal	Huang B and Gong Z. Study on evapo-geochemical barriers in soils of arid-desert landscapes in Tianshan mountains, Xinjiang. *Acta Pedologica Sinica* 2004; **41**: 161-9.
38	English	Journal	Huang B, Gong Z and Gu G. Elemental geochemistry of alto-cryic soils of Qinghai-Tibet Plateau in China-An example from the unpopulated Kekexili region. *Geochemical Journal* 2006; **40**: 211-8.
39	Chinese	Journal	Huang B, Wang J and Jin H *et al.* Effects of long-term application fertilizer on carbon storage in calcareous meadow soil. *Journal of Agro-Environment Science* 2006; **25**: 161-4.
40	Chinese	Journal	Huang D, Lee X and Jiang W *et al.* Geographic variation of carbonate content and pH in surface soil in East Central Asia: Significance as climate proxies. *Geochimica* 2004; **37**: 129-38.
41	Chinese	Journal	Huang P, Zhang J and Zhu A *et al.* Acid and alkaline buffering capacity of typical fluvor-aquic soil in Huang-Huai-Hai Plain. *Scientia Agricultura Sinica* 2009; **42**: 2392-6.
42	Chinese	Journal	Jia Y and Duan J. Spatial distribution characteristics of the carbonate carbon content in soil in a small watershed in the gully region of the Loess Plateau. *Arid Land Geography* 2004; **27**: 166-71.
43	English	Journal	Jiang H, Jiang J and Jia Y *et al.* Soil carbon pool and effects of soil fertility in seeded alfalfa fields on the semi-arid Loess Plateau in China. *Soil Biology & Biochemistry* 2006; **38**: 2350-8.
44	Chinese	Journal	Jiang W, Bai J and Gao H *et al.* Profile distribution characteristics of living elements in wetland soils from typical raised field in Baiyangdian Lake. *Journal of Soil and Water Conservation* 2009; **23**: 261-4.
45	Chinese	Journal	Jin F, Li S and Lu H *et al.* Comparison of the chemical methods for assessing soil N-supplying capacity in calcareous soil. *Plant Nutrition and Fertilizer Science* 2007; **13**: 1040-8.
46	Chinese	Journal	Li B, Wang J and Zhao S *et al.* The influence of fertilizers on the soil fertility, population structure and yield of herbages grown in degraded grassland. *Grassland of China* 2004; **26**: 15-8.
47	English	Journal	Li M, Hou YL and Zhu B. Phosphorus sorption-desorption by purple soils of China in relation to their properties. *Australian Journal of Soil Research* 2007; **45**: 182-9.
48	Chinese	Journal	Li S. Comparison of different methods for the determination of exchangeable base in calcareous soil. *Ningxia Journal of Agriculture and Forestry Science and Technology* 2007; **5**: 46-8.
49	Chinese	Journal	Li S, Zhou J and Wang H *et al.* Characteristics of fixation and release of phosphorus in three soils. *Acta Pedologica Sinica* 2003; **40**: 908-14.

No.	Language	Type	Reference
50	Chinese	Dissertation	Li X. Study on selection of plant materials for soil and water conservation in southern section of the Taihang Mountain. *Ph.D. Thesis*. Beijing Forestry University, 2006.
51	Chinese	Journal	Li X, Chen X and Zhou L *et al.* Soil moisture characteristics and their affecting factors in the rocky desertification process of karst regions, Southwest China. *Journal of Soil and Water Conservation* 2008; **22**: 198-203.
52	Chinese	Journal	Li X, Chen X and Zhou L *et al.* Study on soil organic carbon fractions and their influential factors in rocky desertification process in southwest of China. *Journal of Mountain Science* 2010; **28**: 56-62.
53	Chinese	Journal	Li X, Huang C and Pang J *et al.* Stratigraphy of Holocene palaeoflood slack-water deposits in the Weishui River valley of the western Guanzhong Basin. *Journal of Stratigraphy* 2009; **33**: 198-205.
54	Chinese	Journal	Li X, Wang Z and Hao M *et al.* Evaluation on soil carbon contents under different cropping systems on dryland in Loess Plateau. *Transactions of the CSAE* 2010; **26**: 325-30.
55	Chinese	Journal	Li Y, Bai D and Zhang F *et al.* Effect of acidic rhizosphere fertilizer on the soil pH and Fe availability of calcareous soil. *Plant Nutrition and Fertilizer Science* 2003; **9**: 312-6.
56	Chinese	Journal	Li Y and Chen Z. Research on the current situation of farmland fertility in Tianjin. *Science and Technology of Tianjin Agriculture and Forestry* 2003; **1**: 31-3.
57	Chinese	Journal	Li Z, Wang S and Gao Q *et al.* Effect of Zn and ABT on root growth and the phosphatase and pH at rhizosphere of maize. *Plant Nutrition and Fertilizer Science* 2004; **10**: 156-60.
58	Chinese	Journal	Ling Z, Li Z and Wang S *et al.* The distribution characteristics and paleoclimate significance of the total organic carbon and calcium carbonate of the Kektala profile, Yili, Xinjiang. *Journal of Arid Land Resources and Environment* 2010; **24**: 195-9.
59	Chinese	Journal	Liu J, Chang Q and Zhang J *et al.* Effect of vegetation on soil fertility in different woodlands on Loess Plateau. *Journal of Northwest A&F University (Natural Science Edition)* 2004; **32**: 111-5.
60	English	Journal	Liu L, Shi P and Zou X *et al.* Short-term dynamics of wind erosion of three newly cultivated grassland soils in Northern China. *Geoderma* 2003; **115**: 55-64.
61	Chinese	Journal	Liu M, Chang Q and Yang X. Soil carbon fractions under different land use types in the tablelands of the Loess Plateau. *Plant Nutrition and Fertilizer Science* 2010; **16**: 1418-25.
62	Chinese	Journal	Liu S, Fan W and Wu Z. Studies on relationship between cobalt and phosphorus in calcareous soil. *Journal of Shanxi Agricultural University (Natural Science Edition)* 2004; **4**: 338-41.
63	Chinese	Journal	Liu S, Jie X and Li Y *et al.* Study on bio-availability and transformation of different phosphates in calcareous soils. *Journal of Henan Agricultural University* 2002; **36**: 370-3.
64	Chinese	Journal	Liu S, Zhang S and Wu J *et al.* Relationship between soil pH and calcium carbonate content. *Soils* 2002; **5**: 279-82.
65	Chinese	Journal	Liu W, Du L and Liu D. Study on the transformation and iron provision mechanism of iron fertilizers in the calcareous soil. *Plant Nutrition and Fertilizer Science* 2002; **8**: 344-8.

No.	Language	Type	Reference
66	Chinese	Journal	Liu W and Shang Q. Buffer action and its influencing factors of different types of soil in Changchun Region. *Journal of Jilin Agricultural University* 2001; **23**: 78-82.
67	Chinese	Journal	Liu X and Sun L. Effect of bentonite and phosphorus fertilizer on inorganic phosphorus transformation and their availabilities in calcareous soil. *Acta Agricultutae Nucleatae Sinica* 2003; **18**: 59-62.
68	Chinese	Journal	Liu X, Yan H and Zhang S. Differences of rhizosphere characteristics of two P-efficient wheat genotypes on two calcareous soils. *Soil and Fertilizer Sciences in China* 2009; **4**: 36-9.
69	Chinese	Journal	Liu Z, Liu S and Liu F *et al.* The effects of phosphate on adsorption and desorption of available zinc in calcareous soil. *Journal of Henan Agricultural University* 2005; **39**: 114-8.
70	Chinese	Journal	Long J, Jiang X and Deng Q *et al.* Characteristics of soil rocky desertification in the karst region of Guizhou province. *Acta Pedologica Sinica* 2005; **42**: 419-27.
71	Chinese	Journal	Ma J, Guo L and Liu X. Comparation of the measurement methods of available phosphorus and potassium in calcareous soil of Shanxi. *Chinese Journal of Soil Science* 2006; **37**: 620-1.
72	English	Journal	Ni S and Zhang J. Variation of chemical properties as affected by soil erosion on hillslopes and terraces. *European Journal of Soil Science* 2007; **58**: 1285-92.
73	Chinese	Journal	Ning J, He T and Lin C *et al.* The genetic character and diagnostic characteristic of the soil derived from carbonate rock and its taxonomic classification in Northern Guizhou. *Guizhou Agricultural Sciences* 2009; **37**: 76-81.
74	Chinese	Journal	Pan J and Zhang G. Migration and enrichment of some trace elements in Earth-Cumuli-Orthic Anthro Sols. *Acta Ecologica Sinica* 2009; **46**: 982-90.
75	Chinese	Journal	Pang J, Huang C and Zhang X *et al.* Micromorphological features of the cultivated soil and anthropogenic forest soil at Bailuyuan site, Shaanxi province. *Acta Pedologica Sinica* 2007; **44**: 792-800.
76	Chinese	Journal	Peng H, Xiao H and Wu J *et al.* An indirect method for determination of soil total carbonate. *Soils* 2006; **38**: 477-82.
77	Chinese	Journal	Peng Y, Qian C and Cai X *et al.* Soil enzyme activities in different degraded alpine grassland of Tibet. *Journal of Mountain Science* 2007; **25**: 344-50.
78	Chinese	Journal	Qi G, Tang X and Zuo F *et al.* Application effect of the different phosphate fertilizers on calcareous rice soil. *Journal of Anhui Agricultural Sciences* 2007; **35**: 3616-7.
79	Chinese	Journal	Qi S, Xiao H and Luo F. Soil taxonomy in mountainous areas in the Hexi Region of Gansu Province. *Journal of Mountain Science* 2003; **21**: 763-74.
80	Chinese	Journal	Qi Y and Chang Q. Genetic characteristics and taxonomy on mid-below part of the southern slope of the Riyue mountain. *Journal of Northwest A&F University (Natural Science Edition)* 2004; **32**: 51-4.
81	Chinese	Journal	Qian C, Cai X and Xue H *et al.* Microbes characteristics of soil rehabilitation in Central Tibet. *Journal of Soil and Water Conservation* 2003; **17**: 22-6.
82	Chinese	Journal	Qian C, Cai X and Zhang Y. Effect of rotations on degradation and recovery of dryland soils in Central Tibet. *Journal of Soil and Water Conservation* 2005; **19**: 65-9.

No.	Language	Type	Reference
83	Chinese	Journal	Qiao W, Huo G and Zhang W *et al.* A preliminary study on the evolution of soil quality in Datong Naoshan District of Qinghai province. *Journal of Qinghai Environment* 2008; **2**: 49-52.
84	English	Journal	Qiu S, Ju X and Ingwersen J *et al.* Changes in soil carbon and nitrogen pools after shifting from conventional cereal to greenhouse vegetable production. *Soil & Tillage Research* 2010; **107**: 80-7.
85	Chinese	Dissertation	She D. The characteristics and assessment on soil quality of shrub lands in Loess Plateau. *Ph.D. Thesis*. Northwest A＆F University, 2010.
86	Chinese	Journal	Shi L, Xu F and Liu F *et al.* Study on the allelism of boron efficiency genes in Brassica napus. *Plant Nutrition and Fertilizer Science* 2003; **9**: 229-32.
87	Chinese	Journal	Song F, Zhang M and Yu L. Enrichment and variation of various forms of phosphorus in calcareous vegetable cultivated soils. *Journal of Soil and Water Conservation* 2005; **19**: 67-71.
88	Chinese	Journal	Song X, Zhao Y and Chi G. Study on the explosive technique influence on the physical and chemical properties of chestnut soil. *Journal of Dezhou University* 2005; **21**: 19-22.
89	Chinese	Journal	Su Y, Wang F and Zhang Z *et al.* Soil properties and soil aggregate characteristics in marginal farmlands of oasis in Middle Hexi Corridor Region. *Scientia Agricultura Sinica* 2007; **40**: 741-8.
90	English	Journal	Su Y, Wang X and Yang R *et al.* Effects of sandy desertified land rehabilitation on soil carbon sequestration and aggregation in an arid region in China. *Journal of Environmental Management* 2010; **91**: 2109-16.
91	Chinese	Journal	Sun Y and Xie Q. Genesis of the carbonate in the loess-paleosol-red clay sequence of the Chinese Loess Plateau and its weathering phases. *Bulletin of Mineralogy, Petrology and Geochemistry* 2007; **26**: 170-5.
92	Chinese	Dissertation	Sun Z. The study of soil moisture bearing capacity of stand on slope of west of Shanxi Province. *MSc Thesis*. Beijing Forestry University, 2004.
93	English	Journal	Susanne I, Dirk L and Franz M. Chemical soil properties of reclaimed marsh soil from Zhejiang Province P.R. China. *Geoderma* 2007; **142**: 245-50.
94	Chinese	Journal	Tian X, Hu Z and Li S *et al.* Effects of bicarbonate with identical layer of water and fertilization at the same soil zone on growth and nutrient uptake of maize plants. *Plant Nutrition and Fertilizer Science* 2005; **11**: 757-63.
95	Chinese	Journal	Tian X, Lu X and Mai W *et al.* Effect of calcium carbonate content on availability of zinc in soil and zinc and iron uptake by wheat plants. *Soils* 2008; **40**: 425-31.
96	Chinese	Journal	Tong Y, Shi W and Lv D *et al.* Relationship between soil texture and nitrate distribution and accumulation in three types of soil profile in Shaanxi. *Plant Nutrition and Fertilizer Science* 2005; **11**: 435-41.
97	English	Journal	Wang G, Qian J and Cheng G *et al.* Soil organic carbon pool of grassland soils on the Qinghai-Tibetan Plateau and its global implication. *The Science of the Total Environment* 2002; **291**: 207-17.
98	Chinese	Journal	Wang J, Ma H and Tan H *et al.* Change of carbonate content and record on palaeoclimate fluctuations in Heimahe Loess Section on Southern Qinghai Lake Shore. *Journal of Salt Lake Research* 2005; **13**: 5-8.
99	Chinese	Journal	Wang J, Wang Y and Xu H *et al.* Spatial variability of soil organic matter and calcium carbonate and its reason in Guanzhong farmland. *Agricultural Research in the Arid Areas* 2009; **27**: 23-6.

No.	Language	Type	Reference
100	Chinese	Journal	Wang L, Yi C and Brigitta S *et al.* Genetic characteristics and environmental implications of sedimentary deposits of Lake Nam Co in Tibetan Plateau. *Acta Sedimentologica Sinica* 2009; **27**: 503-10.
101	Chinese	Journal	Wang S, Wu X and Liang D *et al.* Transformation and bioavailability for Pak choi (Brassica chinensis) of different forms of selenium added to calcareous soil. *Acta Scientiae Circumstantiae* 2010; **30**: 2499-505.
102	Chinese	Journal	Wang S and Xia P. Fertilizer efficiency, carryover effect and the effect of phosphorus increment of three kinds of phosphate fertilizers in calcareous fluvaquentic soil. *Chinese Journal of Soil Science* 2010; **41**: 367-72.
103	Chinese	Journal	Wang X, Wei Z and Liu K *et al.* Effects of phosphorus fertilization on the microzone characteristics of a calcareous alluvial soil using incubation method. *Chinese Journal of Soil Science* 2010; **41**: 342-5.
104	Chinese	Journal	Wang Y, Cao J and Zhang X *et al.* Carbonate content and carbon and oxygen isotopic composition of surface soil in the dust source regions of China. *Marine Geology & Quaternary Geology* 2004; **24**: 113-7.
105	English	Journal	Wang Y, Li Y and Ye X *et al.* Profile storage of organic/inorganic carbon in soil: From forest to desert. *Science of the Total Environment* 2010; **408**: 1925-31.
106	English	Journal	Wang Y, Zhang X and Arimoto R *et al.* Characteristics of carbonate content and carbon and oxygen isotopic composition of northern China soil and dust aerosol and its application to tracing dust sources. *Atmospheric Environment* 2005; **39**: 2631-42.
107	Chinese	Dissertation	Wang Z. Effects of crop cultivation and intensive grazing on organic carbon pools and aggregate stability in arid grassland soils. *MSc Thesis*. Gansu Agricultural University, 2006.
108	Chinese	Journal	Wu H, Fan Y and Wang L *et al.* Study on the evaluation of ecological frangibility of the wetlands in the Bosten Lake Region. *Arid Land Geography* 2006; **29**: 287-91.
109	Chinese	Journal	Wu J and Ai L. Soil microbial activity and biomass C and N content in three typical ecosystems in Qilian Mountains, China. *Journal of Plant Ecology (Chinese Version)* 2008; **32**: 465-76.
110	Chinese	Journal	Wu X, Bao J and Yi T *et al.* Extractants and optimum extracting conditions of soil available selenium in calcareous soil. *Journal of Agro-Environment Science* 2009; **28**: 931-6.
111	Chinese	Journal	Wu Y, Tian X and Tong Y *et al.* Assessment of integrated soil fertility index based on principal components analysis. *Chinese Journal of Ecology* 2010; **29**: 173-80.
112	Chinese	Journal	Xia H and Wang K. Effects of soil organic matter on characteristics of phosphorus adsorption and desorption in calcareous yellow fluvo-aquic soil and lime concretion black soil. *Plant Nutrition and Fertilizer Science* 2009; **15**: 1303-10.
113	Chinese	Journal	Xia Y, Lou Y and Yang C *et al.* Characteristics of phosphate adsorption and desportion in paddy soils. *Scientia Agricultura Sinica* 2002; **35**: 1369-74.
114	Chinese	Journal	Xie G, Xu H and Wahafu H. Analysis on numerical distribution of soil organic carbon and calcium carbonate content in Urumqi River Basin. *Xinjiang Agricultural Sciences* 2010; **47**: 780-5.
115	Chinese	Journal	Xie Y, Meng J and Guo L *et al.* Carbonate and carbon isotope characteristics in Harbin sand-dust fallouts. *Geography and Geo-Information Science* 2010; **26**: 63-7.

No.	Language	Type	Reference
116	Chinese	Journal	Xu H and Han B. Soil of formation characteristics and soil vertical distribution in Altay Mountains area, Xinjiang. *Soil and Fertilizer Sciences in China* 2008; **1**: 12-7.
117	Chinese	Journal	Xu W, Zhang Y and Liu H. Preliminary study on the characteristics of ammonia volatilization from salinized soils in Xinjiang. *Ecology and Environment* 2007; **16**: 176-9.
118	Chinese	Journal	Xu Z, Li D and Yang J *et al.* Effect of carbonate on exchangeability and bioavailability of exogenous neodymium in soil. *Journal of the Chinese Rare Earth Society* 2001; **19**: 257-60.
119	Chinese	Journal	Yan D, He T and Ning J *et al.* A preliminary study on the segment characteristic of calcareous paddy soil in karst mountain of Guizhou Province. *Journal of Mountain Agriculture and Biology* 2009; **28**: 109-14.
120	Chinese	Journal	Yan X, Chang Q and Pan J. Classification of Lou Soil in Chinese Soil Taxonomy in Guanzhong Region. *Soils* 2004; **36**: 318-22.
121	Chinese	Journal	Yang N, Jiang Y and Zhao L. Study on soil buffering abilities of different utilization types in saline alkali zone. *Chinese Agricultural Science Bulletin* 2010; **26**: 196-200.
122	Chinese	Dissertation	Yang X. Effects of long-termed in-situ fertilization on rhizosphere nutrition and yield, quality of high gluten wheat in calcareous Chao Soil. *MSc Thesis*. Henan Agricultural University, 2006.
123	Chinese	Journal	Yao X, Ma W and Chu J. Effect of phosphoric acid on pH and micronutrient availability in calcareous soil. *Soils and Fertilizers* 2005; **2**: 14-6.
124	Chinese	Journal	Yao X, Ma W and Chu J. Effects of different acidic materials on pH value of calcareous soil. *Chinese Journal of Eco-Agriculture* 2006; **14**: 68-71.
125	Chinese	Journal	Yi X. Absorption characters and reduction capacities of different Lu Soil layers to Cr(VI). *Agricultural Research in the Arid Areas* 2004; **22**: 215-20.
126	Chinese	Journal	Yu S, Yang L and Jang Q *et al.* Forms, transformation and bioavailability of inorganic phosphorus in calcareous Chao Soil and Cinnamon of Shandong. *Chinese Journal of Soil Science* 2003; **34**: 422-6.
127	Chinese	Journal	Yuan B, Yang J and Lu Y *et al.* Determination of cation exchange capacity in calcareous Purple Soil with the K2C2O4-KCl Double-Quick exchange method. *Journal of Southwest Agricultural University (Natural Science)* 2005; **27**: 914-7.
128	Chinese	Dissertation	Yue Q. Soil effect on vegetation restoration and reconstruction in loess gully and hilly region. *Ph.D. Thesis*. Northwest A＆F University, 2007.
129	Chinese	Journal	Zeng J, Guo T and Bao X *et al.* Effections of soil organic carbon and soil inorganic carbon under long-term fertilization. *Soil and Fertilizer Sciences in China* 2008; **2**: 11-4.
130	Chinese	Dissertation	Zhang J. Vegetation restoration and reconstruction effect on desertified lands of agro-pastoral transition zone of Northern Shannxi. *Ph.D. Thesis*. Northwest A＆F University, 2010.
131	Chinese	Journal	Zhang J, Li X and Qiao X. Analysis on vertical distribution of alpine grassland and meadow in Sanjiangyuan Region. *Hubei Agricultural Sciences* 2009; **48**: 592-6.
132	Chinese	Journal	Zhang L, Sun X and Cao J *et al.* Transfer of soil organic carbon to soil inorganic carbon in carbonate rock soil of desert grassland. *Arid Land Geography* 2010; **33**: 732-9.

No.	Language	Type	Reference
133	Chinese	Journal	Zhang N, He X and Wu W. Studies on the characteristics of soil organic matter and pedogenic calcium carbonate for three kinds of soil in the Tengri Desert. *Acta Ecologica Sinica* 2009; **29**: 4094-101.
134	Chinese	Journal	Zhang Q, Wu W and Wang M *et al.* The effects of crop residue amendent and N rate on soil respiration. *Acta Ecologica Sinica* 2005; **25**: 91-5.
135	Chinese	Journal	Zhang T, Sun R and Hu B *et al.* Analyzing soil carbon characteristics of typical urbanization zones in northwestern Beijing. *Journal of Beijing Normal University (Natural Science)* 2010; **46**: 97-102.
136	Chinese	Journal	Zhang T, Wang Y and Liu J *et al.* Factors influencing swelling of soils in Loess Plateau. *Journal of Northwest A&F University (Natural Science Edition)* 2007; **35**: 185-9.
137	Chinese	Journal	Zhang X, Lian B and Yin J *et al.* Study on influences of different land use types on soil qualities in Plateau Karst Depression. *Journal of Anhui Agricultural Sciences* 2010; **38**: 5771-5.
138	Chinese	Journal	Zhao C and Li L. Composition of stable micro-aggregates in the soil of Lanzhou suburbs. *Journal of Lanzhou University (Natural Sciences)* 2003; **39**: 90-4.
139	Chinese	Journal	Zhao J. The essence and formation model of the loess. *Acta Sedimentologica Sinica* 2003; **21**: 198-204.
140	Chinese	Journal	Zhao R, Ni J and Zhang L *et al.* Effects of carbonates in loess on copper sorption and speciation. *Environmental Chemistry* 2002; **21**: 349-55.
141	Chinese	Dissertation	Zhao Z. The characteristics and assessment of soil fertility of farmland and forestland in the northeast sandy area of Ulanbuh Desert. *MSc Thesis*. Northwest A＆F University, 2010.
142	Chinese	Journal	Zheng Y and Zhang F. Effects of soil moisture and bicarbonate on iron chlorosis of peanut grown on calcareous soil. *Review of China Agricultural Science and Technology* 2000; **2**: 73-6.
143	Chinese	Journal	Zhu A, Zhang J and Zhang Y. Classification and basic properties of Soils in Luancheng County. *Soils* 2003; **35**: 476-80.
144	Chinese	Journal	Zhu H, Zhao C and Li J *et al.* Analysis on respiration of soil in scrub lands and its affecting factors in arid areas. *Arid Land Geography* 2006; **29**: 856-60.
145	English	Journal	Zhu H, Zhao C and Li J *et al.* Analysis of impact factors on scrubland soil respiration in the southern Gurbantunggut Desert, Central Asia. *Environmental Geology* 2008; **54**: 1403-9.

Supplementary Table S7. SIC data sources for the depth function fitting. The sampling depths of these data are greater than 2 m.

No.	Language	Type	Reference
1	Chinese	Journal	Chen Y, Zhang Z and Zhao Y. Distribution of soil carbon in sand-binding area and its relation with soil properties. *Journal of Desert Research* 2017; **37**: 296-304.
2	Chinese	Journal	Deng C, Wang Y and Niu Z *et al.* Effect of land reclamation age on soil physicochemical properties and inorganic carbon in arid areas. *Journal of Soil and Water Conservation* 2017; **31**: 254-9.
3	Chinese	Journal	Dong Y, Cai M and Zhou J. The stocks and characteristics of organic and inorganic carbon in Lou soil in Yangling, Shaanxi. *Journal of Northwest A & F University (Nat. Sci. Ed.)* 2013; **41**: 152-8.
4	English	Journal	Han X, Gao G and Chang R *et al.* Changes in soil organic and inorganic carbon stocks in deep profiles following cropland abandonment along a precipitation gradient across the Loess Plateau of China. *Agriculture, Ecosystems and Environment* 2018; **258**: 1-13.
5	English	Journal	Heimann L, Roelcke M and Hou Y *et al.* Nutrients and pollutants in agricultural soils in the peri-urban region of Beijing: Status and recommendations. *Agriculture, Ecosystems and Environment* 2015; **209**: 74-88.
6	Chinese	Journal	Jia Y and Duan J. The effect of land-use on calcium carbonate in hilly loess soils. *Chinese Journal of Soil Science* 2003; **34**: 319-21.
7	English	Journal	Jin S, Tian X and Wang H. Hierarchical responses of soil organic and inorganic carbon dynamics to soil acidification in a dryland agroecosystem, China. *Journal of Arid Land* 2018; **10**: 726-36.
8	Chinese	Journal	Li X, Huang C and Pang J *et al.* Stratigraphy of Holocene palaeoflood slack-water deposits in the Weishui River valley of the western Guanzhong Basin. *Journal of Stratigraphy* 2009; **33**: 198-205.
9	Chinese	Journal	Ling Z, Li Z and Wang S *et al.* The distribution characteristics and paleoclimate significance of the total organic and calcium carbonate of the Kektala profile, Yili, Xinjiang. *Journal of Arid Land Resources and Environment* 2010; **24**: 195-9.
10	English	Journal	Liu W, Wei J and Cheng J *et al*. Profile distribution of soil inorganic carbon along a chronosequence of grassland restoration on a 22-year scale in the Chinese Loess Plateau. *Catena* 2014; **121**: 321-9.
11	Chinese	Journal	Luo Q, Wang Y and Deng C *et al.* Distribution of inorganic carbon in soil profile and its relationship with soil saline-alkali property in arid area. *Journal of Soil and Water Conservation* 2017; **31**: 240-6.
12	Chinese	Journal	Luo Q, Wang Y and Deng C *et al*. Dynamics of soil carbon storage under different land use years in arid agriculture. *Transactions of the Chinese Society of Agricultural Engineering* 2017; **33**: 287-94.
13	Chinese	Journal	Mao N, Shao M and Huang L. Distribution characteristics and influencing factors of soil carbon profile along toposequences in Liudaogou Watershed. *Journal of Soil and Water Conservation* 2017; **31**: 222-30.
14	English	Journal	Mu C, Zhang T and Zhang X *et al.* Pedogenesis and physicochemical parameters influencing soil carbon and nitrogen of alpine meadows in permafrost regions in the northeastern Qinghai-Tibetan Plateau. *Catena* 2016; **141**: 85-91.

No.	Language	Type	Reference
15	Chinese	Journal	Pan J and Zhang G. Migration and enrichment of some trace elements in Earth-cumuli-orthic Anthrosols. *Acta Pedologica Sinica* 2009; **46**: 982-90.
16	English	Journal	Song BL, Yan MJ and Hou H *et al.* Distribution of soil carbon and nitrogen in two typical forests in the semiarid region of the Loess Plateau, China. *Catena* 2016; **143**: 159-66.
17	English	Journal	Song Y, Yao Y and Qin X *et al.* Response of carbon and nitrogen to afforestation from 0 to 5m depth on two semiarid cropland soils with contrasting inorganic carbon concentrations. *Geoderma* 2020; **357**: 113940.
18	Chinese	Journal	Wang N, Xu W and Xu H *et al.* Spatial variation of soil carbon and stable isotopes in the southern margin desert of Junggar Basin, China. *Chinese Journal of Applied Ecology* 2017; **28**: 2215-21.
19	English	Journal	Wang Y, Jiang J and Niu Z *et al.* Responses of soil organic and inorganic carbon vary at different soil depths after long-term agricultural cultivation in Northwest China. *Land Degradation & Development* 2019; **30**: 1229-42.
20	English	Journal	Wang Y, Li Y and Ye X *et al.* Profile storage of organic/inorganic carbon in soil: From forest to desert. *Science of the Total Environment* 2010; **408**: 1925-31.
21	English	Journal	Wang Y, Wang Z and Li Y. Storage/turnover rate of inorganic carbon and its dissolvable part in the profile of saline/alkaline soils. *PLoS ONE* 2013; **8**: e82029.
22	Chinese	Journal	Yan A, Wang Z and Jiang P *et al.* Effects of soil salinity on vertical distribution of soil carbon in saline soil in arid area. *Arid Zone Research* 2017; **34**: 770-4.
23	Chinese	Journal	Yang W, Zhang S and Yang X. Effect of long-term soil management and fertilization on storage of soil inorganic carbon and its relationship with soil organic carbon content in plough layer of manual loess soil. *Journal of Northwest A & F University (Nat. Sci. Ed.)* 2016; **44**: 74-82.
24	English	Journal	You M, Han X and Hu N *et al.* Profile storage and vertical distribution (0-150 cm) of soil inorganic carbon in croplands in northeast China. *Catena* 2020; **185**: 104302.
25	English	Journal	Yu X, Zhou W and Chen Y *et al.* Spatial variation of soil properties and carbon under different land use types on the Chinese Loess Plateau. *Science of the Total Environment* 2020; **703**: 134946.
26	Chinese	Journal	Zhang BB, Liu F and Ding JZ *et al.* Soil inorganic carbon stock in alpine grasslands on the Qinghai-Xizang Plateau: An updated evaluation using deep cores. *Chinese Journal of Plant Ecology* 2016; **40**: 93-101.
27	English	Journal	Zhang F, Wang X and Guo T *et al.* Soil organic and inorganic carbon in the loess profiles of Lanzhou area: implications of deep soils. *Catena* 2015; **126**: 68-74.
28	Chinese	Journal	Zhang Q, Zhang J and Wang L *et al.* Vertical distribution of soil organic and inorganic carbon in the Taklimakan Desert Highway Shelterbelt drip-irrigated with different mineralization water. *Journal of Northwest Forestry University* 2019; **34**: 1-7.
29	English	Journal	Zhang Y, Xie Y and Ma H *et al.* Rebuilding soil organic C stocks in degraded grassland by grazing exclusion: A linked decline in soil inorganic C. *PeerJ* 2020; **8**: e8986.
30	English	Journal	Zhao W, Zhang R and Huang C *et al.* Effect of different vegetation cover on the vertical distribution of soil organic and inorganic carbon in the Zhifanggou Watershed on the Loess Plateau. *Catena* 2016; **139**: 191-8.

References

1. Raza S, Miao N and Wang P *et al*. Dramatic loss of inorganic carbon by nitrogen-induced soil acidification in Chinese croplands. *Global Change Biol* 2020; **26**: 3738–51.
2. Li ZP, Han FX and Su Y *et al*. Assessment of soil organic and carbonate carbon storage in China. *Geoderma* 2007; **138**: 119–26.
3. Wu H, Guo Z and Gao Q *et al*. Distribution of soil inorganic carbon storage and its changes due to agricultural land use activity in China. *Agr Ecosyst Environ* 2009; **129**: 413–21.
4. Song XD, Wu HY and Ju B *et al.* Pedoclimatic zone-based three-dimensional soil organic carbon mapping in China. *Geoderma* 2020; **363**: 114145.
5. Yang Y, Fang J and Ji C *et al.* Soil inorganic carbon stock in the Tibetan alpine grasslands. *Global Biogeochem Cy* 2010; **24**: GB4022.
6. Zamanian K, Pustovoytov K and Kuzyakov Y. Pedogenic carbonates: Forms and formation processes. *Earth-Sci Rev* 2016; **157**: 1–17.
7. Zhao Y, Wang M and Hu S *et al*. Economics- and policy-driven organic carbon input enhancement dominates soil organic carbon accumulation in Chinese croplands. *Proc Natl Acad Sci USA* 2018; **115**: 4045–50.
8. Tang X, Zhao X and Bai Y *et al.* Carbon pools in China's terrestrial ecosystems: New estimates based on an intensive field survey. *Proc Natl Acad Sci USA* 2018; **115:** 4021–6.

9. Xiong X, Grunwald S and Corstanje R *et al*. Scale-dependent variability of soil organic carbon coupled to land use and land cover. *Soil Till Res* 2016; **160**: 101–9.

10. Li Z, Cao W and Liu B *et al*. Current status and developing trend of soil erosion in China. *Science of Soil and Water Conservation* 2008; **6**: 57–62. (in Chinese with English abstract)

11. Pan GX. Pedogenic carbonates in aridic soils of China and the significance in terrestrial carbon transfer. *Journal of Nanjing Agricultural University* 1999; **22**: 51–7. (in Chinese with English abstract)

12. Wei Y, Liu S and Huntzinger DN *et al*. NACP MsTMIP: Global and North American Driver Data for Multi-Model Intercomparison. Tennessee: Oak Ridge National Laboratory; 2014. http://dx.doi.org/10.3334/ORNLDAAC/1220.

13. Guo JH, Liu XJ and Zhang Y *et al*. Significant acidification in major Chinese croplands. *Science* 2010; **327**: 1008–10.

14. Zhu Q, Vries WD and Liu X *et al*. The contribution of atmospheric deposition and forest harvesting to forest soil acidification in China since 1980. *Atmos Environ* 2016; **146**: 215–22.

15. Yang Y, Ji C and Ma W *et al*. Significant soil acidification across northern China's grasslands during 1980s–2000s. *Global Change Biol* 2012; **18**: 2292–300.

16. Chi W, Zhao Y and Kuang W *et al*. Impacts of anthropogenic land use/cover changes on soil wind erosion in China. *Sci Total Environ* 2019; **668**: 204–15.

17. Zhang XY, Arimoto R and An ZS. Dust emission from Chinese desert sources linked to variations in atmospheric circulation. *J Geophys Res-Atmos* 1997; **102**: 28041–7.
18. McBratney AB, Santos MM and Minasny B. On digital soil mapping. *Geoderma* 2003; **117**: 3–52.
19. Liaw A and Wiener M. Classification and regression by randomForest. *R News* 2002; **2**: 18–22.
20. Balesdent J, Basile-Doelsch I and Chadoeuf J *et al*. Atmosphere–soil carbon transfer as a function of soil depth. *Nature* 2018; **559**: 599–602.
21. Díaz-Hernández JL, Fernández EB and González JL. Organic and inorganic carbon in soils of semiarid regions: a case study from the Guadix–Baza basin (Southeast Spain). *Geoderma* 2003; **114**: 65–80.
22. Song Y, Yao Y and Qin X *et al.* Response of carbon and nitrogen to afforestation from 0 to 5m depth on two semiarid cropland soils with contrasting inorganic carbon concentrations. *Geoderma* 2020; **357**: 113940.
23. Mi N, Wang S and Liu J *et al.* Soil inorganic carbon storage pattern in China. *Global Change Biol* 2008; **14**: 2380–7.
24. Yang Y, Fang J and Ji C *et al*. Widespread decreases in topsoil inorganic carbon stocks across China's grasslands during 1980s–2000s. *Global Change Biol* 2012; **18**: 3672–80.
25. Hengl T, de Jesus JM and Heuvelink GBM *et al.* SoilGrids250m: global gridded soil information based on machine learning. *PLoS One* 2017; **12**: e0169748.

26. Pelletier JD, Broxton PD and Hazenberg P *et al.* A gridded global data set of soil, immobile regolith, and sedimentary deposit thicknesses for regional and global land surface modeling. *J Adv Model Earth Sy* 2016; **8**: 41–65.

27. Yan F, Shangguan W and Zhang J *et al*. Depth-to-bedrock map of China at a spatial resolution of 100 meters. *Sci Data* 2020; **7**: 1–13.

28. Song X, Ge G and Zhu J *et al.* ParallelDSM: Parallel Digital Soil Mapping using Machine Learning. R package version 0.1; 2020.

29. Sanderman J, Hengl T and Fiske GJ. Soil carbon debt of 12,000 years of human land use. *Proc Natl Acad Sci USA* 2017; **114**: 9575–80.

30. Wang T, Yang D and Yang Y *et al.* Permafrost thawing puts the frozen carbon at risk over the Tibetan Plateau. *Sci Adv* 2020; **6**: eaaz3513.

31. Jarvis A, Reuter HI and Nelson A *et al*. Hole-filled SRTM for globe (Version 4). CGIAR-CSI; 2008. http://srtm.csi.cgiar.org

32. O'Neill BC, Tebaldi C and van Vuuren DP *et al*. The Scenario Model Intercomparison Project (ScenarioMIP) for CMIP6. *Geosci Model Dev* 2016; **9**: 3461–82.

33. Fick SE and Hijmans RJ. WorldClim 2: new 1-km spatial resolution climate surfaces for global land areas. *Int J Climatol* 2017; **37**: 4302–15.

34. Hegglin M, Kinnison D and Lamarque JF. CCMI nitrogen surface fluxes in support of CMIP6 (Version 20161207). Earth System Grid Federation; 2016. https://doi.org/10.22033/ESGF/input4MIPs.1125

35. Li X, Yu L and Sohl T *et al.* A cellular automata downscaling based 1 km global land use datasets (2010–2100). *Sci Bull* 2016; **61**: 1651–61.

36. Guo Y, Wang X and Li X *et al.* Dynamics of soil organic and inorganic carbon in the cropland of upper Yellow River Delta, China. *Sci Rep-UK* 2016; **6**: 36105.

37. Tan WF, Zhang R and Cao H *et al.* Soil inorganic carbon stock under different soil types and land uses on the Loess Plateau region of China. *Catena* 2014; **121**: 22–30.

38. Wang Y, Li Y and Ye X *et al*. Profile storage of organic/inorganic carbon in soil: from forest to desert. *Sci Total Environ* 2010; **408**: 1925–31.

39. Chang R, Fu B and Liu G *et al*. The effects of afforestation on soil organic and inorganic carbon: A case study of the Loess Plateau of China. *Catena* 2012; **95**: 145–52.

40. Han X, Gao G and Chang R *et al.* Changes in soil organic and inorganic carbon stocks in deep profiles following cropland abandonment along a precipitation gradient across the Loess Plateau of China. *Agr Ecosyst Environ* 2018; **258**: 1–13.

41. Jin S and Wang H. Relationships between soil pH and soil carbon in China's carbonate soils. *Fresen Environ Bull* 2018; **27**: 605–11.

42. Li C, Li Q and Zhao L *et al.* Land-use effects on organic and inorganic carbon patterns in the topsoil around Qinghai Lake basin, Qinghai-Tibetan Plateau. *Catena* 2016; **147**: 345–55.

43. Liu W, Wei J and Cheng J *et al*. Profile distribution of soil inorganic carbon along a chronosequence of grassland restoration on a 22-year scale in the Chinese Loess Plateau. *Catena* 2014; **121**: 321–9.

44. Liu Y, Dang ZQ and Tian FP *et al*. Soil organic carbon and inorganic carbon accumulation along a 30-year grassland restoration chronosequence in semi-arid regions (China). *Land Degrad Dev* 2017; **28**: 189–98.

45. Shi Y, Baumann F and Ma Y *et al*. Organic and inorganic carbon in the topsoil of the Mongolian and Tibetan grasslands: pattern, control and implications. *Biogeosciences* 2012; **9**: 2287–99.

46. Wang KB, Ren ZP and Deng L *et al*. Profile distributions and controls of soil inorganic carbon along a 150-year natural vegetation restoration chronosequence. *Soil Sci Soc Am J* 2016; **80**: 193–202.

47. Zhao J, Dong Y and Wang Y *et al.* Natural vegetation restoration is more beneficial to soil surface organic and inorganic carbon sequestration than tree plantation on the Loess Plateau of China. *Sci Total Environ* 2014; **485-486**: 615–23.

48. Bughio MA, Wang P and Meng F *et al*. Neoformation of pedogenic carbonates by irrigation and fertilization and their contribution to carbon sequestration in soil. *Geoderma* 2015; **262**: 12–9.

49. Gao Y, Dang P and Zhao Q *et al*. Effects of vegetation rehabilitation on soil organic and inorganic carbon stocks in the Mu Us Desert, northwest China. *Land Degrad Dev* 2018; **29**: 1031–40.

50. Gao Y, Tian J and Pang Y *et al*. Soil inorganic carbon sequestration following afforestation is probably induced by pedogenic carbonate formation in northwest China. *Front Plant Sci* 2017; **8**: 1282.
51. Shi HJ, Wang XJ and Zhao YJ *et al.* Relationship between soil inorganic carbon and organic carbon in the wheat-maize cropland of the North China Plain. *Plant Soil* 2017; **418**: 423–36.
52. Su YZ, Wang XF and Yang R *et al*. Effects of sandy desertified land rehabilitation on soil carbon sequestration and aggregation in an arid region in China. *J Environ Manage* 2010; **91**: 2109.
53. Wang JP, Wang XJ and Zhang J *et al*. Soil organic and inorganic carbon and stable carbon isotopes in the Yanqi Basin of Northwestern China. *Eur J Soil Sci* 2015; **66**: 95–103.
54. Wang X, Wang J and Xu M *et al.* Carbon accumulation in arid croplands of northwest China: pedogenic carbonate exceeding organic carbon. *Sci Rep-UK* 2015; **5**: 11439.
55. Yu P, Li Q and Jia H *et al.* Effect of cultivation on dynamics of organic and inorganic carbon stocks in Songnen Plain. *Agron J* 2014; **106**: 1574–82.
56. Zhang F, Wang X and Guo T *et al*. Soil organic and inorganic carbon in the loess profiles of Lanzhou area: implications of deep soils. *Catena* 2015; **126**: 68–74.
